# Impact and Beneficial Critical Points of Clinical Outcome in Corticosteroid Management of Adult Patients With Sepsis: Meta-Analysis and GRADE Assessment

**DOI:** 10.3389/fphar.2019.01101

**Published:** 2019-09-24

**Authors:** Lu-Lu Lin, Hui-Yun Gu, Jie Luo, Long Wang, Chao Zhang, Yu-Ming Niu, Hong-Xia Zuo

**Affiliations:** ^1^Center for Evidence-Based Medicine and Clinical Research, Taihe Hospital, Hubei University of Medicine, Shiyan, China; ^2^Department of Pathophysiology, School of Basic Medical Sciences of Wuhan University, Wuhan, China; ^3^Department of Orthopedics, Zhongnan Hospital of Wuhan University, Wuhan University, Wuhan, China; ^4^Department of Intensive Care Unit, Taihe Hospital, Hubei University of Medicine, Shiyan, China

**Keywords:** sepsis, septic shock, corticosteroids, long course low-dose, 28-day mortality, dose-response meta-analysis, GRADE

## Abstract

**Background:** With new randomised pieces of evidence and the latest clinical practice guideline from the *BMJ* emerging in 2018, an updated analysis of best available evidence on the controversial effects of corticosteroids in sepsis is warranted.

**Objectives:** To comprehensively evaluate whether corticosteroids are beneficial in reducing mortality and what cumulative dosage, daily dosage, and duration of corticosteroid treatment would enable adult patients with sepsis to reach the critical point of benefits.

**Methods:** Ovid MEDLINE, Ovid EMbase, Cochrane Library, and LILACS database were searched until March 22, 2019.

**Results:** Thirty RCTs with 8,836 participants were identified. Long course low-dose corticosteroid therapy could improve 28-day mortality (RR = 0.90, 95% CI = 0.84–0.97; high quality), intensive care unit mortality (RR = 0.87; 95% CI = 0.79–0.95; moderate quality), and in-hospital mortality (RR = 0.88, 95% CI = 0.79–0.997; high quality). However, we found no benefits for 90-day, 180-day, and 1-year mortality. Subgroup results of long course corticosteroid treatment in a population with septic shock and vasopressor-dependent septic shock, corticosteroid regimen with hydrocortisone plus fludrocortisone, corticosteroid dosing strategies including bolus dosing and infusion dosing, the strategies of abrupt discontinuation, timing of randomisation ≤24 h, impact factor of ≥10, and sample size ≥500 were associated with a marginally reduction in 28-day mortality.

**Conclusions:** This meta-analysis found that the long course low-dose and not short course high-dose corticosteroid treatment could marginally improve short-term 28-day mortality with high quality, especially septic shock and vasopressor-dependent septic shock, and it is recommended that long course (about 7 days) low-dose (about 200–300mg per day) hydrocortisone (or equivalent) with cumulative dose (at least about 1,000mg) may be a viable management option for overall patients with sepsis, and it can be also adapted to patient with septic shock alone. Early hydrocortisone plus fludrocortisone administration, *via* continuous infusion or bolus dosing, is also particularly important for the prognosis. Abrupt discontinuation of corticosteroids, as opposed to the conventional tapered discontinuation, may be considered as a desirable option in 28-day mortality. The safety profile of long course low-dose corticosteroid treatment, including adverse hyperglycaemia and hypernatraemia events, remains a concern, although these events could be easily treated.

**Clinical Trial Registration:** PROSPERO, identifier CRD 42018092849.

## Introduction

Sepsis is characterised by a dysregulated host response to infection, resulting in life-threatening circulatory, cellular, and metabolic abnormalities, and is associated with a substantial burden on global healthcare systems (Bone et al., 1992; Levy et al., 2003; Singer et al., 2016). Data from 730 participating centres in 84 countries in 2012 (Vincent et al., 2014) showed that sepsis contributed to high intensive care unit (ICU) and in-hospital mortality rates. However, patients with septic shock, a subset of sepsis, require a vasopressor to maintain the mean arterial pressure. Septic shock is associated with an extremely high mortality rate of >40% (Singer et al., 2016). Currently, sepsis and septic shock is recognised as a global health priority by the World Health Organization (Reinhart et al., 2017).

Although hydrocortisone was first used for severe infections in the 1960s (Bennett et al., 1963), based on randomised controlled trials (RCTs), the role of corticosteroids in the management of sepsis and septic shock remains highly controversial. Based on the biological mechanisms of sepsis and the pharmacological mechanism of corticosteroids, high-dose corticosteroids (Schumer, 1976; Sprung et al., 1984; Bone et al., 1987; Veterans Administration Systemic Sepsis Cooperative Study Group, 1987; Luce et al., 1988) were adopted to block potential bursts of pro-inflammatory cytokines and suppress inflammation. However, randomised studies (Schumer, 1976; Sprung et al., 1984; Bone et al., 1987; Luce et al., 1988) and previous systematic reviews (Cronin et al., 1995; Lefering and Neugebauer, 1995) on short course high-dose corticosteroid treatment did not report the expected reduction in mortality due to sepsis or septic shock, and reported even worse situations with an increased risk of mortality (Minneci et al., 2009). Subsequently, numerous RCTs (Bollaert et al., 1998; Briegel et al., 1999; Chawla and Tessler, 1999; Annane et al., 2002; Oppert et al., 2005; Sprung et al., 2008; Keh et al., 2016; Lv et al., 2017; Tongyoo et al., 2016) and meta-analyses (Annane et al., 2004a; Annane et al., 2004b; Minneci et al., 2004; Agarwal et al., 2007; Moran et al., 2010; Annane et al., 2015; Volbeda et al., 2015; Rygard et al., 2018) have focused on the benefits and harms of long course low-dose corticosteroid treatment with divergent conclusions. Two unprecedented studies, the ADRENAL (Venkatesh et al., 2018) and APROCCHSS (Annane et al., 2018) trials published in 2018 in the *New England Journal of Medicine*, aimed to put an end to this historical controversy, but the uncertainty with dramatic difference (Suffredini, 2018) remained due to this difference in the number of patients enrolled appears to be related to different inclusion (i.e., high-dose norepinephrine or epinephrine in APROCCHS) and exclusion criteria (e.g., death likely from a pre-existing disease within 90 days in ADRENAL). Subsequent up-date meta-analyses (Fang et al., 2019; Lyu et al., 2018; Ni et al., 2019; Rochwerg et al., 2018; Rygard et al., 2018; Xu et al., 2018; Zhou et al., 2018) with aggregated contradictory points also made this unadorned conflicting conclusion more confusing and intriguing.

Currently, the optimal daily dosage and duration of corticosteroid treatment are also still controversial based on the clinical evidence brought forth by relevant studies (Minneci et al., 2004; Keh and Sprung, 2004; Loisa et al., 2007; Rhodes et al., 2017), which issued differentiated statements that intravenous corticosteroids with certain characteristics are recommended in adult patients with septic shock, who, despite adequate fluid replacement, require vasopressor therapy to maintain adequate blood pressure, and determined whether glucocorticoid administrations at dosages similar to physiological levels during a stressful state affect the outcome in septic patients. However, the latest clinical practice guideline (Lamontagne et al., 2018) published by the *BMJ* in 2018, which also provided an overview of the benefits and harms of corticosteroid therapy in sepsis, also emphasised that the optimal corticosteroid dose and duration of treatment are still uncertain. Similarly, other key controversial issues, including population subtype, types of corticosteroids, dosing and discontinuance strategies of corticosteroids, and timing of randomisation, still exist based on different evidences (Briel et al., 2018; Lamontagne et al., 2018; Meduri et al., 2018).

Together with the conflicting conclusions of previous systematic reviews (Annane et al., 2004a; Annane et al., 2009; Annane et al., 2015; Fang et al., 2019; Lyu et al., 2018; Ni et al., 2019; Rochwerg et al., 2018; Rygard et al., 2018; Xu et al., 2018; Zhou et al., 2018), we performed this meta-analysis involving dose-response meta-analysis with meta-regression and trial sequential analysis (TSA) to investigate the benefits and harms of corticosteroids for sepsis populations. Furthermore, our study also explored the optimal cumulative dose, daily dose, and duration of long course low-dose corticosteroid therapy that can reach the critical point of benefits amongst patients with sepsis, to provide better scientifically strategic guidelines for clinical practice.

## Materials and Methods

This systematic review and meta-analysis was performed according to the Preferred Reporting Items for Systematic reviews and Meta-Analyses (PRISMA) statement (Moher et al., 2009). The protocol for this systematic review and meta-analysis was registered and approved in the International Prospective Register of Systematic Reviews (PROSPERO) under registration number CRD 42018092849.

## Data Sources and Database Searches

We searched Ovid MEDLINE, Ovid Embase, the Cochrane Central Register of Controlled Trials (CENTRAL), and LILACS (Latin American and Caribbean Health Sciences Literature) from their inception to March 22, 2019 for eligible literature focusing on the use of corticosteroids for the treatment of sepsis and septic shock. We also manually searched the reference lists of relevant reviews, meta-analyses, and clinical practice guidelines. The search strategy is described in **Supplementary Method 1**.

## Study Selection

All included studies met the following criteria: 1) population: adult patients (aged ≥16 years) diagnosed with sepsis, septic shock, or any combinations thereof (Bone et al., 1992; Levy et al., 2003; Singer et al., 2016); 2) interventions: administration of any type of corticosteroid, including but not limited to hydrocortisone, methylprednisolone, betamethasone, and dexamethasone; 3) controls: placebo or standard therapy (including antibiotics, fluid replacement, inotropic or vasopressor therapy, mechanical ventilation, or dialysis) without corticosteroids; 4) all studies reporting at least one of the following predefined outcomes: short-term mortality including 14-day and 28-day mortality; long-term mortality including 90-day, 180-day, and 1-year mortality; ICU mortality; in-hospital mortality; number of participants with shock reversal at days 7 and 28; sequential organ failure assessment (SOFA) score at day 7; length of ICU and hospital stay; adverse events including gastrointestinal bleeding, superinfection, hyperglycaemia, hypernatraemia, and neuromuscular weakness; and 5) RCTs published in the English language. Our primary outcomes of interest were 28-day and 90-day mortality during long course low-dose corticosteroid therapy.

## Data Extraction and Quality Assessment

Four authors (H-YG, L-LL, JL, and LW) independently extracted relevant information using a standardised data collection form. Data extracted included lead author’s name, year of study publication, duration of the study, patient characteristics, interventions, controls, and outcomes. For corticosteroid, related information, including types of corticosteroids, length and daily dose of the course, dosing strategies, and discontinuance strategies, should be extracted. As long as any of the available data of defined primary or secondary outcomes were reported, it should be extracted. For any missing data, we first looked for relevant information from previous meta-analyses that included the study or we obtained such data by calculation using existing data where possible. If the suitable data were still not availabled, we will contact the author of the original literature by email to obtain the missing information. Any disagreement during the data extraction stage was resolved by discussion and consultation with a fifth author (**CZ**).

The risk of bias in the included studies was independently assessed by two authors using the Cochrane Collaboration’s tool for assessing the risk of bias (Higgins and Green, 2011). The included studies were assessed based on the following items scored as high, low, and unclear risks: random sequence generation, allocation concealment, blinding of participants and personnel, blinding of outcome assessors, incomplete outcome data, selective reporting, and other sources of bias. Any disagreement was resolved by discussion and consultation with a third author if necessary.

## Assessment of Overall Quality of Study Evidence Using the GRADE Framework

The overall quality of evidences regarding 28-day and 90-day mortality, ICU mortality, and in-hospital mortality was assessed according to the Grading of Recommendations Assessment, Development and Evaluation (GRADE) framework based on five items including the risk of bias, inconsistency, indirectness, imprecision, and publication bias (Guyatt et al., 2008). For each outcome, the quality of evidence was downgraded if the above items were assessed as having serious limitations (Balshem et al., 2011).

## Data Synthesis and Analysis

Meta-analyses were performed using the *meta* package (version 4.9-1) of the R statistical software (version 3.1.1; The R Development Core Team, Vienna, Austria). Dichotomous and continuous outcomes were expressed as relative risks (RR) with 95% confidence intervals (CI) (Deeks, 2002), and mean differences (MD) with 95% CI (Higgins and Green, 2011), respectively, at a significance level of 0.05. Heterogeneity between studies was assessed using the chi-squared test, for which the significance level was set at 0.1, and quantified using the *I* ^2^ statistic (Higgins and Thompson, 2002). *I* ^2^ values of ≥40% were interpreted as indicating significant heterogeneity, and in such cases, a random-effects model was used to conduct the meta-analysis; for *I* ^2^ values of <40%, a fixed-effect model was used instead (Higgins and Green, 2011). Publication bias (Egger et al., 1997; Higgins and Green, 2011) was assessed using Egger’s regression and L’Abbe plot for mortality.

To better reflect the impact of corticosteroids for sepsis and septic shock, we performed subgroup analyses for outcomes based on treatment duration (long and short course) as well as dosage (low- and high-dose) and emphasised the differences between short course high-dose and long course low-dose corticosteroid therapy. Long course treatment was defined as treatment lasting three or more days (Annane et al., 2015); high-dose was defined as a dose of over 400 mg of hydrocortisone per day (or equivalent) (Annane et al., 2015). Dose conversion for corticosteroids in this meta-analysis was performed according to the *Oxford Handbook of Critical Care* (Singer and Andrew, 2009). When doses were presented as milligrams per kilogram body weight, we estimated a body weight of 75 kg. In addition, subgroup analyses were performed based on population subtype [severe sepsis, sepsis and acute respiratory distress syndrome (ARDS), sepsis and community-acquired pneumonia (CAP), septic shock, vasopressor-dependent septic shock, and critical illness- related corticosteroid insufficiency], types of corticosteroids (hydrocortisone, hydrocortisone plus fludrocortisone, dexamethasone, methylprednisolone, and prednisolone), dosing strategies of corticosteroids (bolus, infusion, and bolus plus infusion), discontinuance strategies of corticosteroids (tapered discontinuation and abrupt discontinuation), timing of randomisation (≤24 h and >24 h), baseline severity of disease [Acute Physiology and Chronic Health Evaluation (APACHE) II score <25 and ≥25], definitions for sepsis and septic shock [Sepsis 1.0, 1992 (Bone et al., 1992); Sepsis 2.0, 2001 (Levy et al., 2003); Sepsis 3.0, 2016 (Singer et al., 2016)], sample size (Rucker et al., 2011) (<500 and ≥500), impact factors (IFs) (Littner et al., 2005) of journal based on the year 2017 (<10 and ≥10) when the included study was published, and double-blind study design (Higgins and Green, 2011) for 28-day and 90-day mortality during long course low-dose corticosteroid treatment.

To further explore the factors influencing the pooled effect of corticosteroids, meta-regression analyses were performed according to the APACHE II score, baseline mortality of the control group, sample size, and year of publication of included studies reporting the benefits and harms of long course low-dose corticosteroid therapy for 28-day mortality. To identify how much optimal cumulative dose, daily dose, and duration of long course low-dose corticosteroid therapy with 28-day mortality could reach the critical point of benefits amongst patients with overall population and only septic shock, the dose-response meta-analyses were employed according to cumulative dose (including loading dose and full dose), full dose at study day 1, and time at full dose of hydrocortisone (or equivalent) using the robust error meta-regression model (Xu and Doi, 2018). This is a multilevel log-linear regression model that treats the dose (or time) of corticosteroid as an independent variable and the risk of 28-day mortality as the dependent variable across the entire dataset (Xu et al., 2019). A weighted least squares method was used for the parameter estimation considering the discrepancy of sample size for each study. The robust variance was used to deal with the correlations for the logRR of intervention against the control (logRR = 0). We further explored the impact of baseline severity of disease on the effect of corticosteroids by multiple subgroups based on the different cut-off baseline mortality rates of the control group (Dellinger et al., 2013; Suffredini, 2018), and numbers needed to treat were also calculated for 28-day and 90-day mortality for long course low-dose treatment.

## Trial Sequential Analysis

Similar to interim analysis in a single trial, conventional meta-analysis has a limitation in that sparse data and repeated significance testing could introduce random errors, increasing the risk of type I error (Brok et al., 2009; Wetterslev et al., 2008). In a meta-analysis, the risk of reaching a falsely positive or falsely negative conclusion should be minimised (Sterne and Davey Smith, 2001). TSA is useful for determining whether the evidence in a meta-analysis is reliable and conclusive using conventional monitoring boundaries and trial sequential monitoring boundaries (Pogue and Yusuf, 1998). In addition, inferences on statistical significance should be made concerning the strength of the evidence, which was defined using the required information size (RIS) in TSA (Thorlund et al., 2009; Wetterslev et al., 2009; Thorlund et al., 2011). In this meta-analysis, we calculated a diversity adjusted RIS using α = 0.05 (two-sided), β = 0.10 (power 90%). All TSA were performed using the Copenhagen Trial Unit’s TSA software (version 0.9.5.10 beta).

## Role of the Funding Source

No funding was obtained for this study.

## Results

### Characteristics of Included Trials

**Figure 1:**
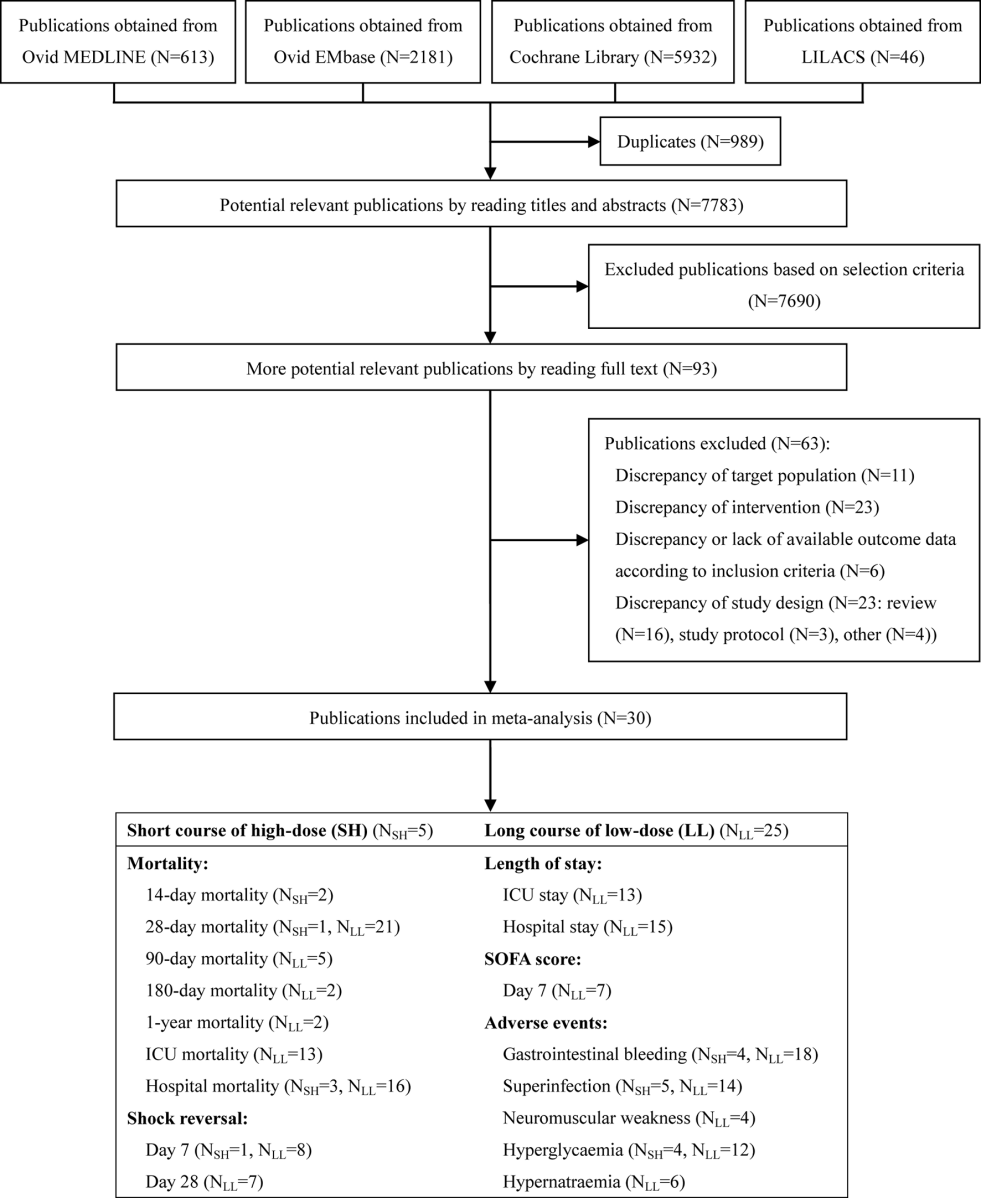
Summary of trial identification and selection. Note: LL, long course of low-dose; SH, short course of high-dose.

Our comprehensive literature search strategy yielded 8,772 results (**Figure 1**). After excluding 8,679 clearly irrelevant records, we obtained full-text articles of the remaining 93 records for further assessment. Eventually, a total of 30 studies (Schumer, 1976; Sprung et al., 1984; Bone et al., 1987; Veterans Administration Systemic Sepsis Cooperative Study Group, 1987; Luce et al., 1988; Bollaert et al., 1998; Briegel et al., 1999; Chawla and Tessler, 1999; Annane et al., 2002; Yildiz et al., 2002; Confalonieri et al., 2005; Oppert et al., 2005; Tandan and Gupta, 2005; Rinaldi et al., 2006; Cicarelli et al., 2007; Meduri et al., 2007; Sprung et al., 2008; Arabi et al., 2010; Snijders et al., 2010; Meijvis et al., 2011; Sabry, 2011; Yildiz et al., 2011; Rezk, 2013; Mirea et al., 2014; Torres et al., 2015; Keh et al., 2016; Tongyoo et al., 2016; Lv et al., 2017; Annane et al., 2018; Venkatesh et al., 2018) enrolling 8,749 participants were included. The reasons for excluding the 63 full-text articles are summarised in **Supplementary Method 2**.

Of the 30 included studies (**Table 1**), 5 studies (Schumer, 1976; Sprung et al., 1984; Bone et al., 1987; Veterans Administration Systemic Sepsis Cooperative Study Group, 1987; Luce et al., 1988) compared the benefit and harm of short course high-dose corticosteroid therapy with placebo, and the remaining 25 studies (Bollaert et al., 1998; Chawla and Tessler, 1999; Briegel et al., 1999; Annane et al., 2002; Yildiz et al., 2002; Confalonieri et al., 2005; Oppert et al., 2005; Tandan and Gupta, 2005; Rinaldi et al., 2006; Cicarelli et al., 2007; Meduri et al., 2007; Sprung et al., 2008; Arabi et al., 2010; Snijders et al., 2010; Meijvis et al., 2011; Sabry, 2011; Yildiz et al., 2011; Rezk, 2013; Mirea et al., 2014; Torres et al., 2015; Keh et al., 2016; Tongyoo et al., 2016; Lv et al., 2017; Annane et al., 2018; Venkatesh et al., 2018) compared long course low-dose corticosteroid therapy with placebo or standard therapy without corticosteroid. All articles were published between 1976 and 2018, and only 11 studies reported the APACHE II score.

**Table 1:** Summary of included clinical trials and patient characteristics.

Study	Year	Duration of study	Number of sites	Population	Sample	APACHE II score	Interventions	Outcomes
Schumer	1976	1967 to 1975	1	Septic shock	172	NA	**Group 1:** Dexamethasone 3mg/kg, a single bolus at the time of diagnosis over 10 to 20 min or methylprednisolone 30mg/kg, a single bolus at the time of diagnosis over 10 to 20 min. Treatment was repeated after 4 hours but not again **Group 2:** Placebo	Hospital mortality; Adverse events
Sprung	1984	August 1979 to February 1982	2	Vasopressor-dependent septic shock	59	NA	**Group 1:** Methylprednisolone 30mg/kg, IV over 10-15 min **Group 2:** Dexamethasone 6mg/kg, IV 10-15 min **Group 3:** No corticosteroid same dose was repeated in four hours, if shock persisted. No further corticosteroid was given after the second dose	28-day mortality; Hospital mortality; Shock reversal by day 7; Adverse events
VASSCSG	1987	NA	10	Severe sepsis, Septic shock	223	NA	**Group 1:** Methylprednisolone 30mg/kg as a single intravenous 10- to 15-min infusion, followed by a constant infusion of 5 mg/kg/h for 9 hours **Group 2:** Placebo	14-day mortality; Adverse events
Bone	1987	November 1982 to December 1985	19	Severe sepsis, Septic shock	382	NA	**Group 1:** Methylprednisolone 30mg/kg 20-min intravenous infusion, every 6 hours for 24 hours **Group 2:** Placebo	14-day mortality; Adverse events
Luce	1988	September 1983 to August 1986	1	Sepsis, ARDS	75	NA	**Group 1:** Methylprednisolone 30mg/kg 15-min intravenous infusion every 6 hours for 24 hours **Group 2:** Placebo	Hospital mortality; Adverse events
Bollaert	1998	NA	2	Vasopressor-dependent septic shock	41	NA	**Group 1:** Hydrocortisone 100mg intravenous bolus every 8 hours for 5 days, then tapered over 6 days **Group 2:** Placebo	28-day mortality; Hospital mortality; ICU mortality; Length of hospital stay; Length of ICU stay; Shock reversal by day 7 and 28; Adverse events
Briegel	1999	NA	1	Vasopressor-dependent septic shock	40	26/27	**Group 1:** Hydrocortisone 100mg 30-min intravenous infusion followed by 0.18 mg/kg/h continuous infusion until shock reversal, then tapered off **Group 2:** Placebo	28-day mortality; 90-day mortality; Hospital mortality; ICU mortality; Length of ICU stay; Shock reversal by day 7 and 28; Adverse events
Chawla	1999	NA	1	Vasopressor-dependent septic shock	44	NA	**Group 1:** Hydrocortisone 100mg intravenous bolus every 8 hours for 3 days, then tapered over 4 days **Group 2:** Placebo	28-day mortality; Hospital mortality; ICU mortality; Length of hospital stay; Length of ICU stay; Shock reversal by day 7 and 28; Adverse events
Yildiz	2002	May 1997 to April 1999	1	Sepsis, Severe sepsis and Septic shock	40	15.4/17.9	**Group 1:** Prednisolone 2 intravenous boluses: 5mg at 06:00 and 2.5 mg at 18:00 for 10 days **Group 2:** Placebo	28-day mortality; Hospital mortality; Length of hospital stay; Adverse events
Annane	2002	October 1995 to February 1999	19	Vasopressor-dependent septic shock	300	NA	**Group 1:** Hydrocortisone 50mg intravenous bolus every 6 hours for 7 days plus fludrocortisone 50μg taken orally every 24 hours for 7 days **Group 2:** Placebo	28-day mortality; 1-year morality; Hospital mortality; ICU mortality; Length of hospital stay; Length of ICU stay; Shock reversal by day 7 and 28; Organ dysfunction at day 7; Adverse events
Tandan	2005	NA	1	Septic shock, Adrenal insufficiency	28	NA	**Group 1:** Hydrocortisone (stated low dose but actual dose and duration not reported) **Group 2:** Placebo	28-day mortality; Hospital mortality; Shock reversal by day 28
Confalonieri	2005	July 2000 to March 2003	6	Sepsis, CAP	46	17.2/18.2	**Group 1:** Hydrocortisone 200mg intravenous loading bolus followed by a continuous infusion at a rate of 10mg/h for 7 days, then tapered over 4 days **Group 2:** Placebo	28-day mortality; 90-day mortality; Hospital mortality; ICU mortality; Length of hospital stay; Length of ICU stay; Adverse events
Oppert	2005	NA	1	Vasopressor-dependent septic shock	40	25/25.5	**Group 1:** Hydrocortisone 50mg of intravenous bolus followed by 0.18mg/kg/h continuous infusion up to cessation of vasopressor for ≥1 hour, reduced to a dose of 0.02mg/kg/h for 24 hours, then reduced by 0.02 mg/kg/h every day **Group 2:** Placebo	28-day mortality; Shock reversal by day 7; Organ dysfunction at day 7
Rinaldi	2006	NA	1	Severe sepsis	52	NA	**Group 1:** Hydrocortisone 300mg per day as a continuous infusion for 6 days, then tapered off **Group 2:** Standard therapy	28-day mortality; Hospital mortality; ICU mortality; Length of ICU stay; Organ dysfunction at day 7
Cicarelli	2007	November 2004 to December 2005	1	Vasopressor-dependent septic shock	29	20/19	**Group 1:** Dexamethasone 0.2mg/kg intravenous, 3 doses at intervals of 36 hours **Group 2:** Placebo	28-day mortality; Organ dysfunction at day 7; Adverse events
Meduri	2007	April 1997 to April 2002	5	Severe sepsis, ARDS	61	NA	**Group 1:** Methylprednisolone loading dose of 1mg/kg followed by continuous infusion of 1mg/kg/d from day 1 to day 14, then 0.5mg/kg/d from day 15 to day 21, then 0.25mg/kg/d from day 22 to day 25, then 0.125mg/kg/d from day 26 to day 28. If participant was extubated before day 14, he/she was advanced to day 15 of drug therapy. Treatment was given intravenously until enteral intake was restored, then was given as a single oral dose **Group 2:** Placebo	28-day mortality; Hospital mortality; ICU mortality; Length of hospital stay; Length of ICU stay; Adverse events
Sprung	2008	March 2002 to November 2005	52	Septic shock	499	NA	**Group 1:** Hydrocortisone 50mg every 6 hours for 5 days, then 50mg every 12 hours for 3 days, then 50mg once a day for 3 days **Group 2:** Placebo	28-day mortality; 1-year morality; Hospital mortality; ICU mortality; Length of hospital stay; Length of ICU stay; Shock reversal by day 7 and 28; Organ dysfunction at day 7; Adverse events
Arabi	2010	April 2004 to October 2007	1	Septic shock, Cirrhosis	75	30.0/29.3	**Group 1:** Hydrocortisone 50mg intravenous bolus every 6 hours until shock resolution, then treatment tapered off by 1 ml every 2 days until discontinuation **Group 2:** Placebo	28-day mortality; Hospital mortality; ICU mortality; Length of hospital stay; Length of ICU stay; Shock reversal by day 7; Organ dysfunction at day 7; Adverse events
Snijders	2010	NA	1	Sepsis, CAP	213	NA	**Group 1:** Prednisolone 40mg intravenous once per day for 7 days **Group 2:** Placebo	28-day mortality; Length of hospital stay; Adverse events
Meijvis	2011	November 2007 to September 2010	2	Sepsis, CAP	304	NA	**Group 1:** Dexamethasone 5mg intravenous bolus once a day for 4 days **Group 2:** Placebo	28-day mortality; Hospital mortality; Length of hospital stay; Length of ICU stay; Adverse events
Sabry	2011	NA	3	Sepsis, CAP	80	NA	**Group 1:** Hydrocortisone intravenous loading dose of 200mg over 30 min, followed by 300mg in 500 mL 0.9% saline at a rate of 12.5 mg/h for 7 days **Group 2:** Placebo	ICU mortality; Shock reversal by day 7; Organ dysfunction at day 7; Adverse events
Yildiz	2011	April 2005 to May 2008	1	Sepsis, Severe sepsis, Septic shock	55	22.9/18.7	**Group 1:** Prednisolone intravenous 3 times a day at 06:00 (10 mg), 14:00 (5 mg) and 22:00 (5mg) for 10 days **Group 2:** Placebo	28-day mortality; Adverse events
Rezk	2013	NA	1	Sepsis, ARDS	27	NA	**Group 1:** Methylprednisolone loading dose of 1mg/kg followed by infusion of 1mg/kg/d from day 1 to day 14, 0.5mg/kg/d from day 15 to day 21, 0.25mg/kg/d from day 22 to day 25 and 0.125mg/kg/d from day 26 to day 28 **Group 2:** Placebo	Adverse events
Mirea	2014	NA	1	Septic shock	181	NA	**Group 1:** 200mg/day hydrocortisone hemisuccinate in four doses or continuous administration; **Group 2:** Placebo	Length of hospital stay
Torres	2015	NA	3	Sepsis, CAP	61	NA	**Group 1:** Methlyprednisolone intravenous bolus of 0.5mg/kg/12 h for 5 days started within 36 hours of hospital admission **Group 2:** Placebo	Hospital mortality; ICU mortality; Length of hospital stay; Length of ICU stay
Keh	2016	January 2009 to August 2013	34	Severe sepsis	353	19.5/18.5	**Group 1:** Hydrocortisone intravenous bolus of 50mg, followed by a 24-hour continuous infusion of 200mg on 5 days, 100 mg on days 6 and 7, 50mg on days 8 and 9, and 25mg on days 10 and 11 **Group 2:** Placebo	28-day mortality; 90-day mortality; 180-day mortality; Hospital mortality; ICU mortality; Length of hospital stay; Length of ICU stay; Adverse events
Tongyoo	2016	December 2010 to December 2014	1	Severe sepsis, Septic shock, ARDS	197	21.7/21.9	**Group 1:** Hydrocortisone intravenous bolus 50mg in 10ml of normal saline every 6 h for 7 days **Group 2:** Placebo	28-day mortality; Adverse events
Lv	2017	September 2015 to September 2016	1	Septic shock	118	25.5/21.3	**Group 1:** Hydrocortisone continuous infusion 200mg/d for 6 days, and then tapered off. Once all vasopressors were discontinued, half dose for three days, then quarter dose for three days and then stopped **Group 2:** Placebo	28-day mortality; Hospital mortality; Length of hospital stay; Length of ICU stay; Shock reversal by day 28
Annane	2018	September 2008, and June 2015	34	Vasopressor-dependent septic shock	1241	NA	**Group 1:** Hydrocortisone 50-mg intravenous every 6 hours, and fludrocortisone a 50-μg tablet *via* a nasogastric tube once daily in the morning for 7 days **Group 2:** Placebo	28-day mortality; 90-day mortality; 180-day mortality; ICU mortality; Adverse events
Venkatesh	2018	March 2013 to April 2017	69	Vasopressor-dependent septic shock	3713	24/23	**Group 1:** Hydrocortisone 200mg/day: continuous intravenous infusion over a period of 24 hours for a maximum of 7 days or until ICU discharge or death **Group 2:** Placebo	28-day mortality; 90-day mortality; Length of hospital stay; Adverse events

### Risk of Bias

As shown in **Supplementary Figure 1**, nine studies (30%) included at least one low risk item. One study (3.3%) was assessed as high risk for random sequence generation. Two studies (6.7%) were assessed as high risk for allocation concealment, blinding, incomplete outcome data, and other forms of bias. Three studies (10%) were assessed as high risk for selective reporting.

## Benefits of Corticosteroids

### Short-Term Mortality

#### 14-Day and 28-Day Mortality

**Figure 2:**
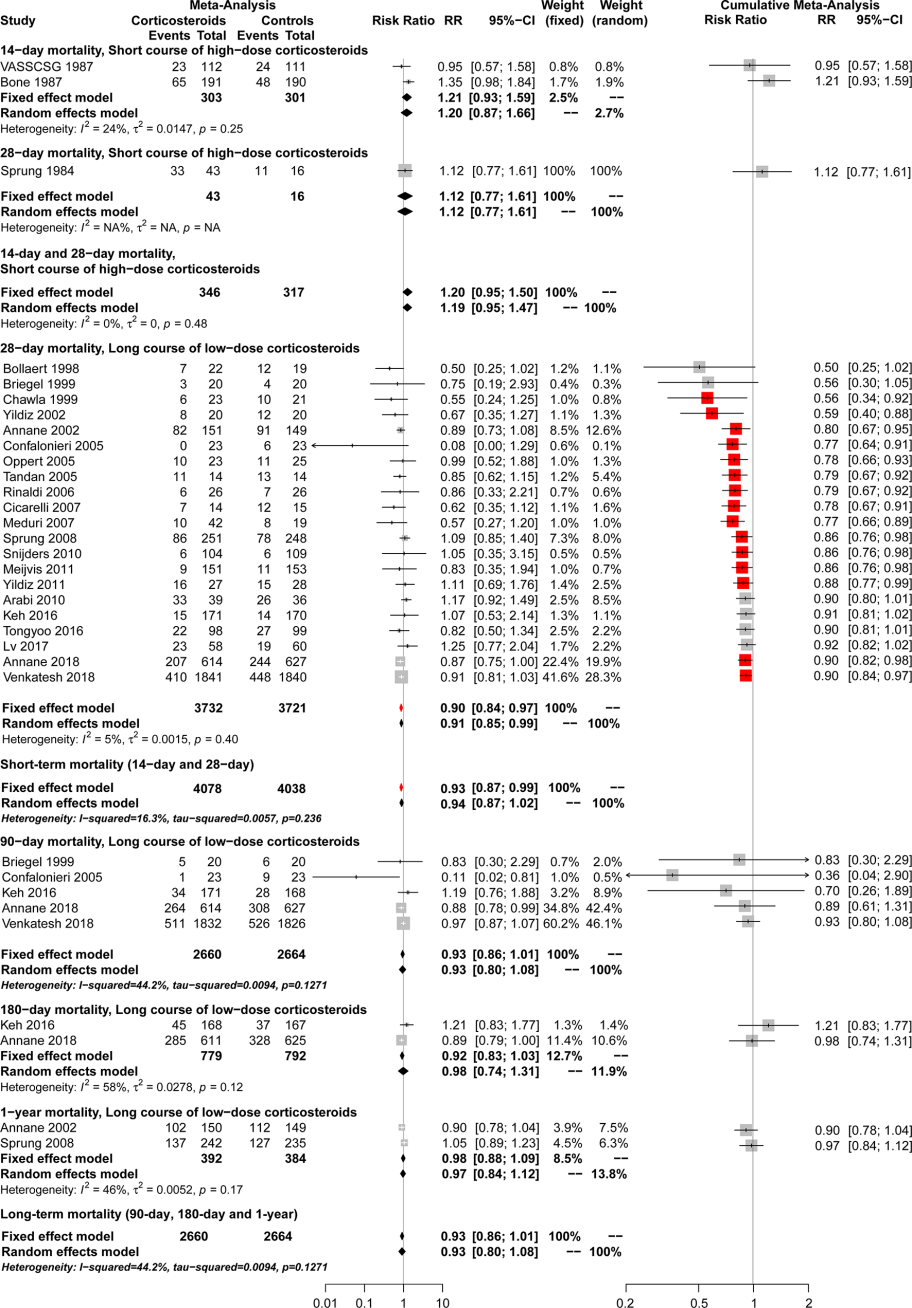
Forest plot of the benefits of corticosteroids for short-term mortality (14-day and 28-day mortality) and long-term mortality (90-day, 180-day and 1-year mortality) from conventional and cumulative analysis.

Data on 14-day mortality were available from 2 studies (Bone et al., 1987; Veterans Administration Systemic Sepsis Cooperative Study Group, 1987). A total of 22 studies (Sprung et al., 1984; Chawla and Tessler, 1999; Bollaert et al., 1998; Briegel et al., 1999; Annane et al., 2002; Yildiz et al., 2002; Confalonieri et al., 2005; Oppert et al., 2005; Tandan and Gupta, 2005; Rinaldi et al., 2006; Meduri et al., 2007; Cicarelli et al., 2007; Sprung et al., 2008; Arabi et al., 2010; Snijders et al., 2010; Meijvis et al., 2011; Yildiz et al., 2011; Keh et al., 2016; Tongyoo et al., 2016; Lv et al., 2017; Venkatesh et al., 2018) reported 28-day mortality rate. Compared with controls, short course high-dose corticosteroid treatment showed no significant results in 14-day and 28-day mortality (RR, 1.20; 95% CI, 0.95–1.50; *I* ^2^ = 0.0%), 14-day mortality (RR, 1.21; 95% CI, 0.93–1.59; *I* ^2^ = 24.0%), and 28-day mortality [RR, 1.12; 95% CI, 0.77–1.61; *I* ^2^ = not applicable (NA)] (**Figure 2**). However, long course low-dose corticosteroid treatment led to a marginal reduction in 28-day mortality rate (RR, 0.90; 95% CI, 0.84–0.97; *I* ^2^ = 5.0%). Regarding the overall short-term mortality (**Figure 2**), including 14-day and 28-day mortality, corticosteroid treatment showed slight significant results (RR, 0.93; 95% CI, 0.87–0.99; *I*^2^ = 16.3%), regardless of length and dose of the course.

#### Subgroup Analyses

**Figure 3:**
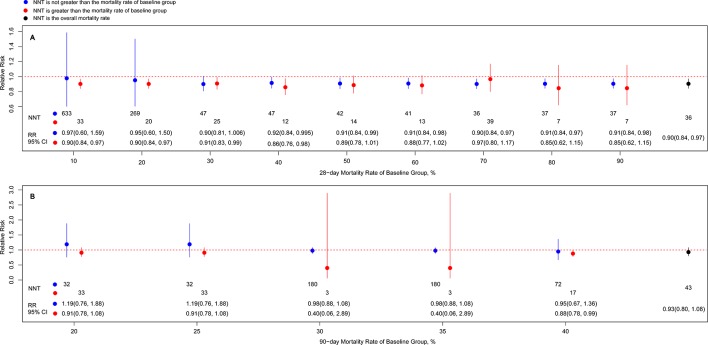
Associations of risk ratio of death at day 28 **(A)**/day 90 **(B)** of long course of low-dose corticosteroids and different cutoff of baseline mortality. Note: Blue node and line indicates the risk ratio (RR) with 95% CI from subgroup based on not greater than the mortality rate of baseline group, and the lower value corresponds to the number needed to treat value; red node and line indicates the RR with 95% CI from subgroup based on greater than the mortality rate of baseline group, and the lower value corresponds to the number needed to treat value; black node and line indicates the RR with 95% CI from overall mortality rate, and the lower value corresponds to the number needed to treat value.

A marginally significant reduction in 28-day mortality was observed in the population with septic shock (RR, 0.91; 95% CI, 0.85–0.99; *I*^2^ = 23.3%) and vasopressor-dependent septic shock (RR, 0.88; 95% CI, 0.81–0.96; *I* ^2^ = 0.0%) (**Table 2**). However, other populations showed no significant difference in 28-day mortality between the long course low-dose corticosteroid treatment group and controls. **Table 2** shows that a slight significant reduction in 28-day mortality was observed with the use of hydrocortisone plus fludrocortisone (RR, 0.87; 95% CI, 0.77–0.98; *I* ^2^ = 0.0%), but not with other corticosteroids. In the subgroup results of dosing strategies for corticosteroid treatment, bolus dosing (RR, 0.92; 95% CI, 0.85–0.99; *I* ^2^ = 0.0%) and infusion dosing (RR, 0.56; 95% CI, 0.38–0.83; *I* ^2^ = 0.0%) showed a reduction in 28-day mortality (**Table 2**), but bolus plus infusion dosing strategies did not. Regarding subgroup analysis of discontinuance strategies for corticosteroids (**Table 2**), abrupt discontinuance (RR, 0.89; 95% CI, 0.82–0.97; *I* ^2^ = 0.0%) was associated with a mild reduction in 28-day mortality, but tapered discontinuance was not. This indicated a slight reduction in 28-day mortality with the timing of randomisation ≤24 h (RR, 0.90; 95% CI, 0.82–0.99; *I* ^2^ = 0.0%), but not with the timing of randomisation >24 h. Significant results for 28-day mortality were not observed among patients with different APACHE II scores (≤25 and >25) (**Table 2**). The subgroup analyses based on definitions for sepsis and septic shock showed marginally significant results for Sepsis 1.0, 1992 (RR, 0.75; 95% CI, 0.56–0.996; *I* ^2^ = 0.0%) and Sepsis 3.0, 2016 (RR, 0.90; 95% CI, 0.82–0.99; *I* ^2^ = 0.0%) (**Table 2**), but not for Sepsis 2.0, 2001. A marginally significant difference was observed in the subgroup of large studies (sample size ≥500) (RR, 0.90; 95% CI, 0.82–0.99; *I* ^2^ = 0.0%), unlike studies with a sample size <500 (**Table 2**). In terms of journal IFs, the benefits of corticosteroids for improving 28-day mortality were reported in studies with IF ≥10 (RR, 0.91; 95% CI, 0.84–0.99; *I* ^2^ = 0.0%; **Table 2**), but not for studies with IF <10. A mild significant difference was observed among double-blind studies (RR, 0.90; 95% CI, 0.84–0.97; *I* ^2^ = 0%) (**Table 2**), but not among unblinded studies. In addition, corticosteroids were not superior to controls when the 28-day mortality rate of the control group was >50% or ≤30%. Furthermore, the analysis according to numbers needed to treat showed a dramatic decrease between the 20% and 30% mortality rates in the control group (**Figure 3**).

**Table 2:** Subgroup analysis of the benefits of long course of low-dose corticosteroids for 28-day and 90-day mortality.

Subgroups	28-day Mortality					90-day Mortality				
	N	Sample	RR, 95% CI	P	I^2^	N	Sample	RR, 95% CI	P	I^2^
**Overall**	21	3732/3721	0.90 (0.84, 0.97)	0.006	5.0%	5	3040/3083	0.93 (0.80, 1.08)	0.34	44.2%
**Population subtype**										
Septic shock	12	3070/3074	0.91 (0.85, 0.99)	0.02	23.3%	3	2466/2473	0.93 (0.86, 1.01)	0.09	0.0%
Sepsis and CAP	3	278/285	0.67 (0.36, 1.26)	0.21	35.9%	1	23/23	0.11 (0.02, 0.81)	0.03	NA
Severe sepsis	2	197/196	0.996 (0.57, 1.75)	0.99	0.0%	1	171/168	1.19 (0.76, 1.88)	0.45	NA
Severe sepsis and ARDS	2	140/118	0.75 (0.50, 1.13)	0.17	0.0%	0	0	NA	NA	NA
Sepsis, severe sepsis and septic shock	2	47/48	0.91 (0.62, 1.32)	0.62	36.3%	0	0	NA	NA	NA
Vasopressor-dependent septic shock	8	2708/2716	0.88 (0.81, 0.96)	0.003	0.0%	3	2466/2473	0.93 (0.86, 1.01)	0.09	0.0%
Critical illness-related corticosteroid insufficiency	9	325/326	0.93 (0.79, 1.09)	0.72	0.0%	1	171/168	1.19 (0.76, 1.88)	0.45	NA
**Types and regimens of corticosteroids**										
Hydrocortisone	13	2609/2601	0.93 (0.85, 1.02)	0.12	19.5%	4	2046/2037	0.95 (0.67, 1.36)	0.78	45.4%
Hydrocortisone plus fludrocortisone	2	765/776	0.87 (0.77, 0.98)	0.03	0.0%	1	614/627	0.88 (0.78, 0.99)	0.03	NA
Dexamethasone	2	165/168	0.72 (0.43, 1.22)	0.23	0.0%	0	0	NA	NA	NA
Methylprednisolone	1	20/20	0.57 (0.27, 1.20)	0.14	NA	0	0	NA	NA	NA
Prednisolone	3	151/157	0.93 (0.65, 1.35)	0.72	0.0%	0	0	NA	NA	NA
**Dosing strategies of corticosteroids**										
Bolus	16	3620/3634	0.92 (0.85, 0.99)	0.02	0.0%	4	2640/2644	0.93 (0.79, 1.10)	57.9%	0.41
Infusion	3	78/53	0.56 (0.38, 0.83)	0.004	0.0%	0	0	NA	NA	NA
Bolus plus Infusion	1	20/20	0.75 (0.19, 2.93)	0.68	NA	1	20/20	0.83 (0.30, 2.29)	0.0%	0.72
**Discontinuance strategies of corticosteroids**										
Taper discontinue	11	698/667	0.97 (0.83, 1.13)	0.67	32.5%	3	1875/1869	0.70 (0.32, 1.56)	0.39	57.2%
Abrupt discontinue	9	3020/3040	0.89 (0.82, 0.97)	0.005	0.0%	2	785/795	0.95 (0.73, 1.23)	0.67	41.3%
**Timing of randomisation**										
≤24 h	6	2212/2209	0.90 (0.82, 0.99)	0.04	0.0%	1	1832/1826	0.97 (0.87, 1.07)	0.54	NA
>24 h	1	23/21	0.55 (0.24, 1.25)	0.15	NA	0	0	NA	NA	NA
**APACHE II score**										
<25	8	2252/2255	0.91 (0.82, 1.01)	0.07	13.9%	3	1184/1230	0.86 (0.51, 1.44)	0.56	0.0%
≥25	3	82/81	1.09 (0.84, 1.39)	0.53	0.0%	2	860/805	1.02 (0.91, 1.16)	0.72	67.6%
**Definitions for sepsis and septic shock**										
Sepsis 1.0, 1992	6	138/134	0.75 (0.56, 0.996)	0.047	0.0%	1	20/20	0.83 (0.30, 2.29)	0.72	NA
Sepsis 2.0, 2001	6	693/688	1.03 (0.90, 1.17)	0.67	0.0%	1	171/168	1.19 (0.76, 1.88)	0.45	NA
Sepsis 3.0, 2016	2	2455/2467	0.90 (0.82, 0.99)	0.02	0.0%	2	1832/1826	0.93 (0.86, 1.01)	0.09	36.6%
Not reported	7	446/432	0.73 (0.56, 0.94)	0.16	0.0%	1	23/23	0.11 (0.02, 0.81)	0.03	NA
**Sample size**										
<500	19	1277/1254	0.92 (0.82, 1.02)	0.12	11.5%	3	214/211	0.71 (0.26, 1.89)	0.49	64.8%
≥500	2	2455/2467	0.90 (0.82, 0.99)	0.02	0.0%	2	2446/2453	0.93 (0.84, 1.02)	0.13	36.6%
**Impact factor**										
<10	13	412/387	0.88 (0.75, 1.02)	0.09	23.7%	1	20/20	0.83 (0.30, 2.29)	0.72	NA
≥10	8	3306/3319	0.91 (0.84, 0.99)	0.02	0.0%	4	2640/2644	0.93 (0.79, 1.10)	0.42	57.9%
**Blinding**										
Double-blind study	19	3667/3659	0.90 (0.84, 0.97)	0.004	0.0%	5	3040/3083	0.93 (0.80, 1.08)	0.34	44.2%
Unblinded study	2	65/62	1.11 (0.85, 1.44)	0.45	0.0%	0	0	NA	NA	NA

ACCP, American College of Chest Physicians; APACHE, Acute Physiology and Chronic Health Evaluation; ARDS, Acute respiratory distress syndrome; ATS, American Thoracic Society; CAP, community-acquired pneumonia; CI, confidence interval; ESICM, European Society of Intensive Care Medicine; NA, Not applicable; SCCM, Society of Critical Care Medicine; SIS, Surgical Infection Society. Sepsis 1.0, The ACCP/SCCM Consensus Conference Committee in 1992; Sepsis 2.0, The SCCM/ESICM/ACCP/ATS/SIS International Sepsis Definitions Conference in 2001; Sepsis 3.0, The third international consensus definitions for sepsis and septic shock by the ESICM/SCCM in 2016.

#### Meta-Regression and Dose-Response Meta-Analyses

No significant results were observed for the interaction of 28-day mortality with APACHE II score (P = 0.053), baseline mortality rate (P = 0.961), year of publication (P = 0.902), and sample size (P = 0.690), using meta-regression (**Supplementary Figure 2**).

**Figure 4:**
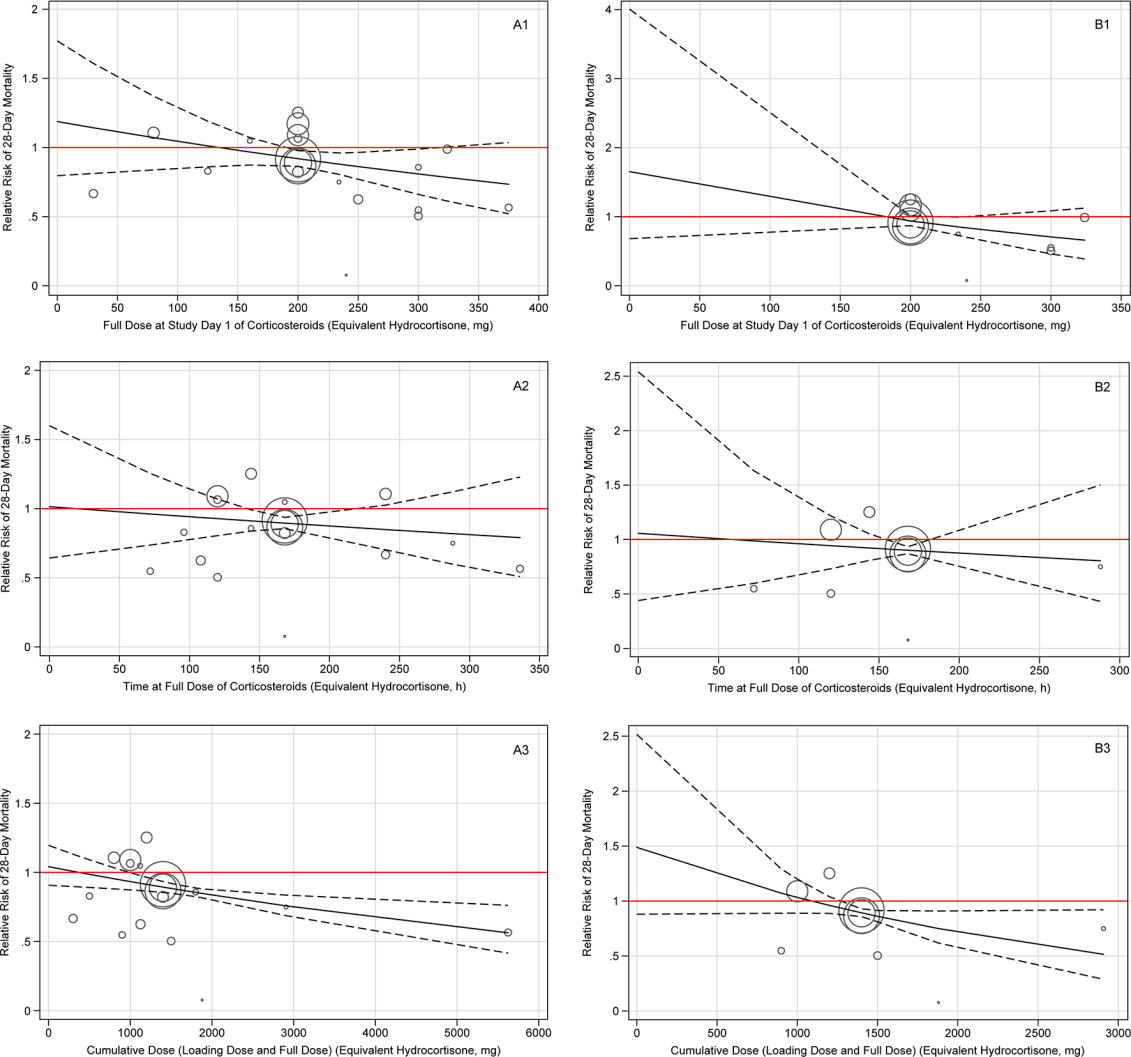
Dose-response of long course of low-dose corticosteroids for 28-day mortality amongst patients with overall patient **(A)** and septic shock alone patient **(B)**. Note: Plate A indicates the overall patient, and plate B indicates the septic shock alone patient. Each plate contains three dose-response relationships, which are the benefits and harms of 28-day mortality and long course of low-dose corticosteroids dose/duration, including full dose at study day 1 (1), time at full dose (2), and cumulative dose (3). The cumulative dose represents the sum of loading dose and full dose. The solid line represents the regression line of the dose-response, and the dashed line represents the 95% confidence interval.

In overall population results of the dose-response meta-analyses shown in **Figure 4A**, long course low-dose corticosteroid treatment showed beneficial effects on 28-day mortality when the cumulative dose of hydrocortisone reached the cutoff value of 1,000 mg (RR, 0.93; 95% CI, 0.78–1.00), and the benefits were sustained as the cumulative dose increased. When measured by full dose at study day 1, the optimal full dose at study day 1 for hydrocortisone with beneficial effects ranged from approximately 200 to 300 mg (200 mg: RR, 0.92; 95% CI, 0.86–0.98; 324 mg: RR, 0.78; 95% CI: 0.61–1.00). When measured by time at full dose, a significant beneficial effect was observed for about 7 days (144 h: RR, 0.91; 95% CI: 0.86–1.00; 168 h: RR, 0.90; 95% CI, 0.86–0.94). Overall, there was a dose-response relationship and a decreasing trend on the risk of death existed as the doses or durations of corticosteroid treatment increased. Further, it is also found that long course (about 7 days of time at full dose) low-dose (approximately 240 mg per day of full dose at study day 1) hydrocortisone (or equivalent) with cumulative dose (at least about 1,400 mg) may be a viable management option for patients with only septic shock (**Figure 4B**).

#### Cumulative Meta-Analyses, TSA, and GRADE

Cumulative analysis showed that results became significant between long course low-dose corticosteroid treatment and controls in 28-day mortality when studies from Chawla and Tessler (1999) and Yildiz et al. (2011) were added. However, significant differences were observed again when the studies of Venkatesh et al. (2018) (ADRENAL trial) and Annane et al. (2018) (APROCCHSS trial) were added (**Figure 2**).

As shown in **Supplementary Figures 3**–**5**, the TSA results showed that the cumulative Z-curve crossed both the conventional boundary and the trial sequential monitoring boundary for benefit, establishing sufficient and conclusive evidence and suggesting further trials were not required for 28-day mortality using long course low-dose corticosteroid therapy [relative risk reduction (RRR) 13%, power 90%; RRR 10%, power 80% for the sensitivity analysis]. In addition, although the cumulative Z-curve did not cross the trial sequential monitoring boundary, it crossed the conventional boundary and RIS line with a 13% 9RRR and 90% power in the sensitivity analysis, suggesting that further trials were not required (**Supplementary Figure 4**).

The overall evidence of long course low-dose corticosteroid treatment for 28-day mortality was assessed to be of high quality using the GRADE assessment framework (**Table 3**).

**Table 3:** GRADE profile of long course of low-dose corticosteroids for sepsis and septic shock in 28-day and 90-day mortality, ICU mortality, and hospital mortality.

Quality assessment						Summary of findings			Quality of evidence
Outcomes	Risk of bias	Inconsistency	Indirectness	Imprecision	Publication bias	Study event rates			
						With corticosteroids	With control	Relative risk (95% CI)	
**28-day Mortality**	No serious limitations	No serious limitations	No serious limitations	No serious limitations	No serious limitations	977/3732 (26.2%)	1074/3721 (28.9%)	0.90 (0.84, 0.97)	?⊕⊕⊕ **High**
**90-day Mortality**	No serious limitations	Serious limitations due to the inconsistency^a^	No serious limitations	Serious limitations due to the imprecision^b^	No serious limitations	815/2660 (30.6%)	877/2664 (32.9%)	0.93 (0.80, 1.08)	⊕⊕○○ **Low**, Due to the inconsistency and imprecision
**Intensive Care Unit Mortality**	No serious limitations	No serious limitations	No serious limitations	No serious limitations	Serious limitations due to the publication bias^c^	483/1463 (33.0%)	546/1445 (37.8%)	0.87 (0.79, 0.95)	⊕⊕⊕○ **Moderate**, Due to the publication bias
**Hospital Mortality**	No serious limitations	No serious limitations	No serious limitations	No serious limitations	No serious limitations	596/1685 (35.4%)	654/1663 (38.7%)	0.90 (0.83, 0.97)	⊕⊕⊕⊕ **High**

^a^The test for heterogeneity is significant, and the I^2^ is moderate, 44%; ^b^95% CI for absolute effects include clinical benefit and no benefit; ^c^Based upon the publication bias test, there is apparent asymmetry for intensive care unit mortality, P = 0.017.

### Long-Term Mortality

#### Mortality



A total of five studies (Briegel et al., 1999; Confalonieri et al., 2005; Keh et al., 2016; Annane et al., 2018; Venkatesh et al., 2018) reported 90-day mortality associated with long course low-dose corticosteroid treatment. No significant superiority of long course low-dose corticosteroid treatment compared with controls was found in reducing 90-day mortality (RR, 0.93; 95% CI, 0.80–1.08; *I* ^2^ = 44.2%) (**Figure 2**).

#### Subgroup Analyses

Subgroup analyses showed that long course low-dose corticosteroid treatment could improve 90-day mortality among patients with sepsis and CAP (RR, 0.11; 95% CI, 0.02–0.81; *I* ^2^ = NA) (**Table 2**). However, other populations showed no significant difference in 90-day mortality between the corticosteroid and control groups. In the subgroup analyses according to type of corticosteroid, hydrocortisone plus fludrocortisone could improve 90-day mortality (RR, 0.88; 95% CI, 0.78–0.99; *I* ^2^ = NA), whereas hydrocortisone alone did not show statistical significance. Furthermore, there were no significant differences between the long course low-dose corticosteroid treatment and control groups in the subgroup analyses based on dosing strategies and discontinuance strategies of corticosteroids, timing of randomisation, APACHE II score, sample size, IFs of the journals, definitions for sepsis and septic shock, and blinding (**Table 2**). **Figure 3** also showed no statistically significant improvement in 90-day mortality among patients who received long course low-dose corticosteroid treatment, only that the cut-off baseline mortality rate was greater than 40%.

#### Cumulative Meta-Analyses, TSA, and GRADE

Cumulative analysis showed a lack of significance between long course low-dose corticosteroid treatment and control groups in 90-day mortality when studies were added (**Figure 2**). The results of TSA showed that the cumulative Z-curve did not cross any boundary, and an additional 85,175 participants (RRR 10%, power 90%), 16,627 participants (RRR 20%, power 90% in the sensitivity analysis), 69,229 participants (RRR 10%, power 80% in the sensitivity analysis) were required to arrive at a firm conclusion (**Supplementary Figures 6**–**8**). In addition, GRADE assessment showed low-quality evidence in 90-day mortality (**Table 3**).

#### 180-day and 1-year Mortality

Long course low-dose corticosteroid treatment led to no improvement in reducing 180-day mortality according to 2 studies (Keh et al., 2016; Annane et al., 2018) (RR, 0.98; 95% CI, 0.74–1.31; *I* ^2^ = 58%) or 1-year mortality according to 2 studies (Annane et al., 2002; Sprung et al., 2008) (RR, 0.97; 95% CI, 0.84–1.12; *I* ^2^ = 46%) (**Figure 2**). Regarding overall long-term mortality, including 90-day, 180-day, and 1-year mortality (**Figure 2**), long course low-dose corticosteroids showed no significant results (RR, 0.93; 95% CI, 0.80–1.08; *I* ^2^ = 44.2%).

Cumulative analysis showed no significant results between long course low-dose corticosteroid and control groups regarding 180-day and 1-year mortality when studies were added (**Figure 2**).

#### ICU Mortality and In-Hospital Mortality

As shown in **Supplementary Figure 9**, long course low-dose corticosteroid treatment could improve ICU mortality (RR, 0.87; 95% CI, 0.79–0.95; *I* ^2^ = 30%). The results of cumulative analysis became significant between long course low-dose corticosteroid and control groups in ICU mortality when the studies of Annane et al. (2002) to Meduri et al. (2007) were added. However, changes in statistical difference were observed again when the studies of Torres et al. (2015), Keh et al. (2016), and Annane et al. (2018) (APROCCHSS trial) were added (**Supplementary Figure 9**). In the TSA, the cumulative Z-curve crossed both the conventional boundary and trial sequential monitoring boundary for benefit, requiring no additional trials (**Supplementary Figures 10** and **11**). Furthermore, the evidence of ICU mortality was of moderate quality in the GRADE assessment (**Table 3**).

**Supplementary Figure 12** also showed that corticosteroids could improve in-hospital mortality (RR, 0.88; 95% CI, 0.79–0.997; *I* ^2^ = 43%). The subgroup results for long course low-dose corticosteroid treatment could improve in-hospital mortality (RR, 0.90; 95% CI, 0.83–0.97; *I* ^2^ = 10%), whereas short course high-dose corticosteroid treatment could not (RR, 0.72; 95% CI, 0.33–1.60; *I* ^2^ = 89%). In addition, the cumulative analysis showed significant results between the long course low-dose corticosteroid and control groups regarding in-hospital mortality when the studies of Chawla and Tessler, (1999) to Meduri et al. (2007), Torres et al. (2015), and Annane et al. (2018) (APROCCHSS trial) were added. The results became significant between long course low-dose corticosteroid treatment and control groups regarding in-hospital mortality when the study of Schumer (1976) (Schumer, 1976) was added (**Supplementary Figure 12**). TSA showed that more trials were required for in-hospital mortality (**Supplementary Figures 13** and **14**). Furthermore, the evidence regarding in-hospital mortality during long course low-dose corticosteroid treatment was of high quality in the GRADE assessment (**Table 3**).

#### Shock Reversal by days 7 and 28

As shown in **Supplementary Figure 15**, corticosteroids could contribute to shock reversal by day 7 (RR, 1.38; 95% CI, 1.25–1.53; *I* ^2^ = 0%). A similar result was observed in the subgroup of long course low-dose corticosteroid treatment (RR, 1.38; 95% CI, 1.25–1.53; *I* ^2^ = 4.5%). However, there were no significant results between the short course high-dose corticosteroid treatment and control groups (RR, 1.55; 95% CI, 0.78–3.06; *I* ^2^ = NA) (**Supplementary Figure 15**). Cumulative analysis showed that significant differences remained between the long course low-dose corticosteroid and control groups when studies were added.

As shown in **Supplementary Figure 16**, long course low-dose corticosteroid treatment could contribute to shock reversal by day 28 (RR, 1.12; 95% CI, 1.02–1.22; *I* ^2^ = 11%). The cumulative analysis yielded significant results that remained significant when the study of Chawla and Tessler (1999) was added.

#### Length of ICU stay and hospital stay

Long course low-dose corticosteroid treatment did not contribute to improvement of the length of ICU stay (MD, −0.73; 95% CI, −2.94 to 1.49; *I* ^2^ = 68.1%) and hospital stay (MD, 0.02; 95% CI, −0.89 to 0.92; *I* ^2^ = 23%) (**Supplementary Figures 17** and **18**). Cumulative analysis showed significant results when data regarding the length of ICU stay from the studies of Confalonieri et al. (2005) to Sprung et al. (2008) were added (**Supplementary Figure 17**). The results were not significant between long course low-dose corticosteroid treatment and control groups regarding the length of hospital stay when studies were added (**Supplementary Figure 18**).

#### SOFA Score at Day 7

A total of seven studies reported SOFA score at day 7 (Annane et al., 2002; Oppert et al., 2005; Rinaldi et al., 2006; Cicarelli et al., 2007; Sprung et al., 2008; Arabi et al., 2010; Sabry, 2011). As shown in **Supplementary Figure 19**, long course low-dose corticosteroid treatment was associated with improved SOFA score at day 7 (MD, −1.83; 95% CI, −2.10 to −1.56; *I* ^2^ = 30.0%). Cumulative analysis showed significant results for long course low-dose corticosteroid treatment when studies were added.

#### Harms of Corticosteroids

Corticosteroids were not found to be associated with a significant increase in gastroduodenal bleeding (**Supplementary Figure 20**), superinfection (Supplementary Figure 21), and neuromuscular weakness (**Supplementary Figure 22**), regardless of the dose or length of course, even in the cumulative meta-analysis. However, long course low-dose corticosteroid treatment led to increased incidences of hyperglycaemia (RR, 1.20; 95% CI, 1.09–1.32; *I* ^2^ = 40%, **Supplementary Figure 23**) and hypernatraemia (RR, 1.66; 95% CI, 1.34–2.06; *I* ^2^ = 0.0%, **Supplementary Figure 24**). Cumulative analysis showed significant results for long course low-dose treatment when data regarding hyperglycaemia and hypernatraemia from the study of Annane et al. (2002) were added.

#### Publication Bias

No obvious publication biases were noted regarding 28-day mortality (P = 0.151), 90-day mortality (P = 0.531), and in-hospital mortality (P = 0.087). However, obvious publication bias was observed regarding ICU mortality (P = 0.017) (**Supplementary Figure 25**).

## Discussion

Sepsis and septic shock constitute a great burden on the global healthcare system (Singer et al., 2016; Reinhart et al., 2017). The evidence of corticosteroids as adjunctive therapy remained controversial. Currently, both the ADRENAL (Venkatesh et al., 2018) and APROCCHSS (Annane et al., 2018) trials showed no beneficial effect on short-term mortality. However, results regarding long-term mortality were significantly different (Suffredini, 2018). The latest meta-analysis (Fang et al., 2019; Lyu et al., 2018; Ni et al., 2019; Rochwerg et al., 2018; Rygard et al., 2018; Xu et al., 2018; Zhou et al., 2018) with aggregated contradictory points also failed to reach a consensus on this disagreement. Meanwhile, the latest clinical practice guideline (Lamontagne et al., 2018) published in *BMJ* in 2018 stated that the optimal corticosteroid dose and duration of treatment are still uncertain. Therefore, we performed a comprehensive updated meta-analysis to assess the impact of corticosteroids and explore how much optimal cumulative dosage, daily dosage, and duration of long course low-dose corticosteroid treatment can reach the critical point of benefits for sepsis and septic shock to guide clinical practice.

Regarding overall short-term mortality, conventional meta-analysis, cumulative meta-analysis, and TSA all confirmed that long course low-dose corticosteroid treatment was beneficial for sepsis and septic shock and short course high-dose corticosteroid treatment was not. More specifically, firstly, long course low-dose corticosteroid treatment could improve 28-day mortality, which further confirmed the results of previous meta-analyses (Annane et al., 2004a; Minneci et al., 2004; Annane et al., 2009; Annane et al., 2015; Fang et al., 2019; Ni et al., 2019). This meta-analysis showed that corticosteroids could improve the short-term mortality (14-day and 28-day) even without subgroup analyses based on duration and dose. Even so, the dose and duration of corticosteroid treatment remain as two important factors influencing the effect of corticosteroids in future studies and in clinical practice. Related studies have demonstrated that short course high-dose corticosteroid treatment had no benefits and even increased the incidence of adverse events among patients with sepsis and septic shock (Sprung et al., 1984; Bone et al., 1987; Luce et al., 1988; Cronin et al., 1995; Minneci et al., 2004; Lefering and Neugebauer, 1995).

With an expanding body of evidence focusing on long course low-dose corticosteroid treatment, we observed the overall benefits in short-term mortality. Although previous studies (Minneci et al., 2004; Loisa et al., 2007; Dellinger et al., 2013) gave different opinions on the recommended dose, the latest clinical guidelines (Lamontagne et al., 2018) published in 2018 again stated that the optimal corticosteroid dose and duration of treatment are still uncertain, which makes the issue of dose even more confusing. To clarify this confusion, dose-response meta-analyses were employed for overall population, which confirmed that long course (about 7 days) low-dose (about 200–300 mg per day) hydrocortisone treatment with cumulative dose (at least about 1,000 mg) was beneficial for the reduction of 28-day mortality. However, it is noteworthy that the dose-response meta-analyses also showed that the over 320 mg per day and time of full dose >10 days yielded no benefit in the 28-day mortality, which deserves attention in the clinical practice guideline (Annane et al., 2017; Lamontagne et al., 2018). Although the similar conclusion was found in sub-population of septic shock, the result based on a small amount of data need to be interpreted with caution. At the same time, we noted that the 90-day mortality reported in the latest meta-analysis in 2018 (Fang et al., 2019) was consistent with our finding. The insignificant pooled results of short-term (within 90 days) mortality obtained by Rygard et al. (2018) was caused by the mixed data of short-term (within 90 days) mortality, which were not separated from those of specific endpoints (14, 28, and 90 days); it may also be due to the truly insignificant results from separate endpoint (90 days). The inconsistent results of mortality due to corticosteroids are the main source of scepticism regarding the use of corticosteroids. In fact, corticosteroids did have benefits for sepsis and septic shock in 28-day mortality.

In addition, for patient subgroups, compared with the controls in this meta-analysis, long course low-dose corticosteroid treatment did not reduce the 28-day mortality among patients with severe sepsis, sepsis and ARDS, sepsis and CAP, and critical illness-related corticosteroid insufficiency, consistent with the 2018 (Lamontagne et al., 2018) clinical practice guideline and latest meta-analysis (Fang et al., 2019), but contrary to the opinions of other clinical guidelines (Annane et al., 2017; Pastores et al., 2018), as in ARDS and CAP patients. However, corticosteroids were superior to controls in terms of reducing 28-day mortality outcomes in septic shock and vasopressor-dependent septic shock patients, which is consistent with the Ger-Inf-05 (Annane et al., 2002) and APROCCHSS (Annane et al., 2018) trials, but contrary to the findings of the latest meta-analysis (Fang et al., 2019). Regarding the types and regimens of corticosteroids, although the 2018 clinical practice guideline (Lamontagne et al., 2018) concluded that adding an agent that has additional mineralocorticoid activity (such as fludrocortisone) could be helpful, but that is highly speculative, our meta-analysis found that the use of hydrocortisone plus fludrocortisone reduced 28-day mortality, and are therefore reasonable choices (Fang et al., 2019; Rygard et al., 2018). When we considered the dosing strategies of corticosteroids, we found that both bolus and infusion strategies could reduce 28-day mortality contrary to the findings of the latest meta-analysis (Rygard et al., 2018), and the infusion seemed to be better. However, it is noteworthy that the recommendation to administer corticosteroids *via* continuous infusion was removed from the 2016 Surviving Sepsis guidelines (Rhodes et al., 2017), and bolus dosing of corticosteroids remains common in clinical practice, which is entirely different from our recommendation. A review of the 2012 Surviving Sepsis guidelines (Dellinger et al., 2013; Hoang et al., 2017) showed that the administration of corticosteroids *via* continuous infusion has been associated with a decreased frequency of hyperglycaemia compared with bolus dosing and is listed as a Grade 2D recommendation. This conclusion made us more confident to recommend infusion dosing of corticosteroids in clinical practice; of course, bolus dosing of corticosteroids should also be retained. However, the combination strategy of bolus plus infusion did not show substantial efficacy, which may be mainly due to its mere pool in a small sample study. The decision to taper or abruptly discontinue corticosteroids is still uncertain with low-quality evidence. It is believed that tapering (Sprung et al., 2008) of corticosteroids could help mitigate adverse immunologic and hemodynamic rebound effects, and the abrupt discontinuation (Keh et al., 2003) of hydrocortisone has been associated with an increase in proinflammatory mediators and hemodynamic instability. However, the abrupt discontinuation of hydrocortisone appears to be safe since it did not produce a rebound effect in the Ger-Inf-05 (Annane et al., 2002), ADRENAL (Venkatesh et al., 2018), and APROCCHSS (Annane et al., 2018) trials. It is noteworthy that when discussing the impact of the discontinuance strategies of corticosteroids on 28-day mortality, we found that tapered discontinuation was actually ineffective in reducing mortality, but abrupt discontinuation can reduce the risk of mortality, an unexpected phenomenon that deserves more attention. Regarding the timing of randomisation, we found that short course of corticosteroids (≤24 h) has a preponderant benefit in reducing 28-day mortality, contrary to the findings of the latest meta-analysis (Rygard et al., 2018). Because of this, the delayed initiation of hydrocortisone (Venkatesh et al., 2018) treatment and time to administration of appropriate antimicrobial therapy (Kumar et al., 2006; Kumar et al., 2009) influences mortality. Although the two subgroups showed differences in reducing 28-day mortality when the cut-off APACHE II score was set at 25, we retained the APACHE II score as an important measure of disease severity. Based on population differences in the definitions of sepsis and septic shock, our study showed that populations from Sepsis 1.0 (1992) and Sepsis 3.0 (2016) confirmed the reduction of 28-day mortality, but that of Sepsis 2.0 (2001) did not. We attribute this difference to the effect of the large sample size being broken, but it was not clear whether the three versions (Bone et al., 1992; Levy et al., 2003; Singer et al., 2016) were affected by the deviation in the definition of the populations. Considering that most results became significant when studies with small sample size were added to the cumulative analysis, we performed subgroup analyses based on sample size, journal IFs, and unblinded study, and found that significant results were more frequently reported in studies with large samples sizes (≥500), high IFs (≥10), and double-blind studies. This also means that a large sample of high quality guarantees the reliability of the results of our study. The results of the meta-regression analyses also roughly confirmed that confounding factors did not influence the results of our study. Upon further analysis of mortality rates of the control group, this meta-analysis demonstrated that corticosteroids could not improve 28-day mortality among patients with baseline mortality rates >50% or ≤30%, which may be valuable for clinical practice.

Regarding long-term mortality, long course low-dose corticosteroid therapy could not improve 90-day, 180-day, and 1-year mortality. Possible reasons were long-term functional disabilities with significant health care and social implications (Iwashyna et al., 2010) and small sample size regarding long-term mortality. The ADRENAL (Venkatesh et al., 2018) and APROCCHSS (Annane et al., 2018) trials conducted in 2018 also had different results regarding 90-day mortality. The reasons may differ by baseline severity of illness and rates of surgical admission, renal-replacement therapy, blood infection, pulmonary infection, urinary tract infection, and abdominal infection (Suffredini, 2018) in the ADRENAL (Venkatesh et al., 2018) and APROCCHSS (Annane et al., 2018) trials. Only sepsis and CAP reduced 90-day mortality, and other populations were not affected. It is noteworthy that the effect of hydrocortisone alone was not significant, but hydrocortisone plus fludrocortisone treatment in the APROCCHSS trial (Annane et al., 2018) yielded statistical differences in 90-day mortality. This differential performance may be triggered due to the fact that the ADRENAL (Venkatesh et al., 2018) and APROCCHSS (Annane et al., 2018) trials had differing conclusions, and both new clinical trials (Annane et al., 2018; Venkatesh et al., 2018) will substantively alter the evidence suggesting a small but uncertain 90-day mortality reduction. At the same time, the clinical practice guidelines (Lamontagne et al., 2018) still have reservations regarding conservative recommendations. Most other subgroup analyses, including the baseline mortality rates, did not show pronounced differences between corticosteroids and controls.

Secondly, in this meta-analysis, long course low-dose corticosteroid treatment could improve ICU and in-hospital mortality, consistent with the result of the latest meta-analysis (Annane et al., 2015). Furthermore, long course low-dose corticosteroid treatment could not shorten the length of ICU stay and hospital stay from conventional analysis and cumulative analysis, contrary to the findings of previous studies (Annane et al., 2009; Annane et al., 2015). Long course low-dose corticosteroid treatment could improve organ dysfunction according to SOFA score at day 7 and contribute to shock reversal by days 7 and 28.

Thirdly, long course low-dose corticosteroid treatment was not found to trigger the adverse events of gastroduodenal bleeding, superinfection, and neuromuscular weakness but increased the incidence of hyperglycaemia and hypernatraemia, consistent with the findings of the study conducted by Fang et al. (Fang et al., 2019). However, previous studies (Sprung et al., 1984; Bone et al., 1987; Minneci et al., 2004) reported that increased mortality caused by short course high-dose corticosteroid treatment might arise from immunosuppressive effects, which would increase the severity of secondary infections. However, this meta-analysis did not find an association of short course high-dose corticosteroid treatment with more events of superinfection and other adverse events including gastroduodenal bleeding and hyperglycaemia.

Interestingly, data from individual studies all failed to show any significant differences between the long course low-dose corticosteroid treatment and control groups regarding the outcome of 28-day mortality. However, the pooled data of all individual studies provided the converse conclusion. This phenomenon may indicate that the sample size of previous individual studies all did not reach the required sample size of those studies, which could reflect significant results. Large-scale RCTs are labour-intensive and require a wealth of resources. Therefore, we conducted a cumulative meta-analysis not only to determine the reliability of the evidence (Haapakoski et al., 2015), but also to reflect whether subsequent studies are able to reverse current conclusions and reduce waste (Clarke et al., 2014). For 28-day mortality, significant results alternated with insignificant results. When the ADRENAL (Venkatesh et al., 2018) and APROCCHSS (Annane et al., 2018) trials were added, the results became significant. We predicted that the results would reach the stable status if subsequent studies would be added in the future. However, the results of TSA demonstrated that the sample size for 28-day mortality was enough (10% RRR, 80% power) and further trials were not required (10% or 13% RRR, 90% power). A similar explanation could be given for ICU mortality.

TSA is a useful tool to detect whether previous studies reached statistical significance and the required sample size (Thorlund et al., 2011). The current analysis showed that more trials were required for 90-day mortality (additional 16,627 participants) and in-hospital mortality (additional 9,498 participants), but not for 28-day mortality and ICU mortality. The RRR and power are two factors associated with results. Therefore, we chose different values of RRR and power based on clinical experience in the sensitivity analysis; a 10% RRR would usually be considered important if the outcome is mortality, but it may not be considered important if the outcome is nausea (Thorlund et al., 2011). The actual achieved power was defined as 1–β (Wetterslev et al., 2008). When the power increases, β also referred to as the type II error decreases. Therefore, a large sample size is required for studies with high power. This theory could explain why the RIS was different based on different values of power and the same value of RRR in our TSA.

This meta-analysis has several strengths. Firstly, conventional meta-analysis, dose-response meta-analysis, meta-regression analysis, cumulative meta-analysis, and TSA were all used in this study. They confirmed each other and enhanced the strength of the evidence. Previous studies including RCTs and meta-analyses provided conflicting conclusions. Our meta-analysis, with an updated evidence base, confirmed the previous positive results. Secondly, compared with previous studies, we updated the evidence regarding long course (about 7 days) low-dose (about 200–300 mg per day) hydrocortisone (or equivalent) treatment with cumulative dose (at least about 1,000 mg) for better management of overall population of sepsis and septic shock in 28-day mortality, and it can be also adapted to patient with septic shock alone. Thirdly, we first provided the over 50% or under 30% mortality rates of the control group as the worst choice of corticosteroids, and if the mortality rates of control group could be converted to mortality rates at baseline, corticosteroids could be used for patients as early as possible to improve survival rate for sepsis and septic shock and guide the clinical practice. However, limitations for this meta-analysis are worth mentioning. First, the number of included studies reporting 90-day mortality was small. Although negative results for long-term mortality including 90-day, 180-day, and 1-year mortality were obtained in this meta-analysis, the true effect of corticosteroids for long-term mortality remains to be investigated in subsequent studies (Suffredini, 2018). Secondly, a small number of studies reported APACHE II score. This meta-analysis failed to find the optimal range of APACHE II score among patients for obtaining benefits from corticosteroid treatment. Thirdly, all the studies fitted to the dose-response model only contained a single dose level compared to the control, and most studies of corticosteroid doses were concentrated at 200–300 mg per day of full dose (hydrocortisone, or equivalent). For this reason, it is insufficient to investigate the potential non-linear dose-response relationship between dose or duration of corticosteroid treatment and 28-day mortality. This is the reason why we only establish a linear relationship between them. At present, a large body of evidence has been gathered to support the short-term outcomes, but more RCTs should be conducted to settle this point and improve the survival rate and quality of life in long-term outcomes.

## Conclusions

This meta-analysis found that the long course low-dose and not short course high-dose corticosteroid treatment could marginally improve short-term 28-day mortality with high quality, especially septic shock and vasopressor-dependent septic shock, and it is recommended that long course (about 7 days of time at full dose) low-dose (about 200–300 mg per day of full dose at study day 1) hydrocortisone (or equivalent) with cumulative dose (at least about 1,000 mg) may be a viable management option for overall patients with sepsis, and it can be also adapted to patient with septic shock alone. Early hydrocortisone plus fludrocortisone administration, *via* continuous infusion or bolus dosing, is also particularly important for the prognosis. Abrupt discontinuation of corticosteroids, as opposed to the conventional tapered discontinuation, may be considered as a desirable option in 28-day mortality, and its clinical benefits should be redefined and recognised in clinical practice. Long course low-dose corticosteroid was also beneficial for in-hospital mortality with high quality and ICU mortality with moderate quality. However, corticosteroid showed no benefits in long-term (90-day, 180-day, and 1-year) mortality. The safety profile of long course low-dose corticosteroid treatment, including adverse hyperglycaemia and hypernatraemia events, remains a concern, although these events could be easily treated.

## Transparency

CZ affirms that the manuscript is an honest, accurate, and transparent account of the study being reported; that no important aspects of the study have been omitted; and that any discrepancies from the study as planned have been explained.

## Data Availability

The datasets analyzed in this manuscript are not publicly available. Requests to access the datasets should be directed to CZ. All data is stored in the manuscript.

## Author Contributions

CZ, H-XZ, and Y-MN conceptualized and coordinated the study. CZ drafted the initial protocol. Y-MN developed the search strategy. L-LL, JL, and H-YG screened citations and assessed studies for eligibility. L-LL, JL, and H-XZ extracted data. H-YG and L-LL performed quality assessments. H-YG, H-XZ, and Y-MN provided content expertise in corticosteroids and Sepsis/Septic Shock. L-LL, CZ, and LW performed statistical analyses. CZ and Y-MN provided methodologic expertise in knowledge synthesis and resolved disagreements regarding study eligibility or quality assessments. CZ, Y-MN, H-XZ, and L-LL critically reviewed the manuscript for important intellectual content. All of the authors gave final approval of the version to be published and agreed to be accountable for all aspects of the work.

## Conflict of Interest Statement

The authors declare that the research was conducted in the absence of any commercial or financial relationships that could be construed as a potential conflict of interest.
