# Supplementary material for: Impact and Beneficial Critical Points of Clinical Outcome in Corticosteroid Management of Adult Patients With Sepsis: Meta-Analysis and GRADE Assessment
Source: Front Pharmacol. 2019 Sep 24;10:1101. doi: 10.3389/fphar.2019.01101 (PMC6771229; doi:10.3389/fphar.2019.01101)

**Supplementary Legends of Method and Figure**

**Supplementary Method 1** Detailed search strategy.

**Supplementary Method 2** Reason from exclusion of literature.

**Supplementary Figure 1** Risk of bias assessment.

**Supplementary Figure 2** Meta-regression of the interaction of long course of low-dose corticosteroids in 28-day mortality and APACHE II score (A), mortality rate of baseline (B), the published year (C) and sample size (D).

**Note:** Size of the data markers indicates weight of the study; APACHE, Acute Physiology and Chronic Health Evaluation.

**Supplementary Figure 3** Trial sequential analysis of long course of low-dose corticosteroids for 28-day mortality.

**Note:** A diversity-adjusted information size of 12,082 patients was calculated based on an anticipated relative risk reduction (RRR) of 10% (event proportion of 28.9% in the control arm, α=0.05 (two-sided), β=0.10 (Power 90%)). The blue cumulative Z-curve was constructed using a fixed-effects model and crossed the conventional boundaries and trial sequential monitoring boundaries when trials were included.
**Supplementary Figure 4** Trial sequential analysis of long course of low-dose corticosteroids for 28-day mortality as sensitive analysis.

**Note:** A diversity-adjusted information size of 7,067 patients was calculated based on an anticipated relative risk reduction (RRR) of 13% (event proportion of 28.9% in the control arm, α=0.05 (two-sided), β=0.10 (Power 90%)). The blue cumulative Z-curve was constructed using a fixed-effects model and crossed the conventional boundaries and trial sequential monitoring boundaries when trials were included.
**Supplementary Figure 5** Trial sequential analysis of long course of low-dose corticosteroids for 28-day mortality as sensitive analysis.

**Note:** A diversity-adjusted information size of 9,025 patients was calculated based on an anticipated relative risk reduction (RRR) of 10% (event proportion of 28.9% in the control arm, α=0.05 (two-sided), β=0.10 (Power 80%)). The blue cumulative Z-curve was constructed using a fixed-effects model and crossed the conventional boundaries but not trial sequential monitoring boundaries. However, the blue cumulative z-curve crossed the required information size (RIS) line when trials were included.
**Supplementary Figure 6** Trial sequential analysis of long course of low-dose corticosteroids for 90-day mortality.

**Note:** A diversity-adjusted information size of 90,499 patients was calculated based on an anticipated relative risk reduction (RRR) of 10% (event proportion of 32.9% in the control arm, α=0.05 (two-sided), β=0.10 (Power 90%)). The blue cumulative Z-curve was constructed using a fixed-effects model and did not cross any boundaries when trials were included.

**Supplementary Figure 7** Trial sequential analysis of long course of low-dose corticosteroids for 90-day mortality as sensitive analysis.

**Note:** A diversity-adjusted information size of 21,951 patients was calculated based on an anticipated relative risk reduction (RRR) of 20% (event proportion of 32.9% in the control arm, α=0.05 (two-sided), β=0.10 (Power 90%)). The blue cumulative Z-curve was constructed using a fixed-effects model and did not cross any boundaries when trials were included.

**Supplementary Figure 8** Trial sequential analysis of long course of low-dose corticosteroids for 90-day mortality as sensitive analysis.

**Note:** A diversity-adjusted information size of 74,553 patients was calculated based on an anticipated relative risk reduction (RRR) of 10% (event proportion of 32.9% in the control arm, α=0.05 (two-sided), β=0.10 (Power 80%)). The blue cumulative Z-curve was constructed using a fixed-effects model and did not cross any boundaries when trials were included.

**Supplementary Figure 9** Forest plot of the benefits of long course of low-dose corticosteroids for ICU mortality.

**Supplementary Figure 10** Trial sequential analysis of long course of low-dose corticosteroids for ICU mortality.

**Note:** A diversity-adjusted information size of 5,333 patients was calculated based on an anticipated relative risk reduction (RRR) of 18% (event proportion of 37.1% in the control arm, α=0.05 (two-sided), β=0.10 (Power 90%)). The blue cumulative Z-curve was constructed using a fixed-effects model and crossed the conventional boundaries and trial sequential monitoring boundaries when trials were included.

**Supplementary Figure 11** Trial sequential analysis of long course of low-dose corticosteroids for ICU mortality as sensitive analysis.

**Note:** A diversity-adjusted information size of 3,984 patients was calculated based on an anticipated relative risk reduction (RRR) of 18% (event proportion of 37.1% in the control arm, α=0.05 (two-sided), β=0.10 (Power 80%)). The blue cumulative Z-curve was constructed using a fixed-effects model and crossed the conventional boundaries and trial sequential monitoring boundaries when trials were included.

**Supplementary Figure 12** Forest plot of the benefits of short course of high-dose and long course of low-dose corticosteroids for in-hospital mortality.

**Supplementary Figure 13** Trial sequential analysis of long course of low-dose corticosteroids for in-hospital mortality.

**Note:** A diversity-adjusted information size of 12,846 patients was calculated based on an anticipated relative risk reduction (RRR) of 9% (event proportion of 39.2% in the control arm, α=0.05 (two-sided), β=0.10 (Power 90%)). The blue cumulative Z-curve was constructed using a fixed-effects model and crossed the conventional boundaries and trial sequential monitoring boundaries when trials were included.

**Supplementary Figure 14** Trial sequential analysis of long course of low-dose corticosteroids for in-hospital mortality as sensitive analysis.

**Note:** A diversity-adjusted information size of 9,596 patients was calculated based on an anticipated relative risk reduction (RRR) of 9% (event proportion of 39.2% in the control arm, α=0.05 (two-sided), β=0.10 (Power 80%)). The blue cumulative Z-curve was constructed using a fixed-effects model and crossed the conventional boundaries and trial sequential monitoring boundaries when trials were included.

**Supplementary Figure 15** Forest plot of the benefits of short course of high-dose and long course of low-dose corticosteroids for shock reversal by day 7.

**Supplementary Figure 16** Forest plot of the benefits of long course of low-dose corticosteroids for shock reversal by day 28.

**Supplementary Figure 17** Forest plot of the benefits of long course of low-dose corticosteroids for length of ICU stay.

**Supplementary Figure 18** Forest plot of the benefits of long course of low-dose corticosteroids for length of hospital stay.

**Supplementary Figure 19** Forest plot of the benefits of long course of low-dose corticosteroids for SOFA score at day 7.

**Supplementary Figure 20** Forest plot of harms of short course of high-dose and long course of low-dose corticosteroids for gastroduodenal bleeding.

**Supplementary Figure 21** Forest plot of harms of short course of high-dose and long course of low-dose corticosteroids for superinfection.

**Supplementary Figure 22** Forest plot of harms of long course of low-dose corticosteroids for neuromuscular weakness.

**Supplementary Figure 23** Forest plot of harms of short course of high-dose and long course of low-dose corticosteroids for hyperglycaemia.

**Supplementary Figure 24** Forest plot of harms of long course of low-dose corticosteroids for hypernatraemia.

**Supplementary Figure 25** The L’Abbe plot and Egger’s regression of corticosteroids of long course of low-dose for 28-day mortality (A), 90-day mortality (B), ICU mortality (C) and hospital mortality (D).

**Supplement Method 1** Detailed search strategy.

**1. Search strategy for Ovid MEDLINE(R) Epub Ahead of Print, In-Process & Other Non-Indexed Citations, Ovid MEDLINE(R) Daily, Ovid MEDLINE and Versions(R)** <1946 to 2019 March 22>

#1 exp Sepsis /(115377)

#2 exp Shock, Septic /(21342)

#3 (sepsis or septic shock).mp. /(126560)

#4 #1 or #2 or #3 /(173622)

#5 exp Adrenal Cortex Hormones /(383450)

#6 (corticosteroid* or steroid*).mp. /(395245)

#7 #6 or #5 /(669900)

#8 #4 and #7 /(7265)

#9 ((randomized controlled trial or controlled clinical trial).pt. or randomized.ab. or placebo.ab. or clinical trials as topic.sh. or randomly.ab. or trial.ti.) not (animals not (humans and animals)).sh. /(1108431)

#10 #8 and #9 /(613)

**2. Search strategy for** **Ovid EMbase Classic + EMbase** <1947 to 2019 March 22>

#1 exp Sepsis /(252555)

#2 exp Shock, Septic /(48565)

#3 (sepsis or septic shock).mp. /(233303)

#4 #1 or #2 or #3 /(289456)

#5 exp Adrenal Cortex Hormones /(966500)

#6 (corticosteroid* or steroid*).mp. /(703270)

#7 #6 or #5 /(1280809)

#8 #4 and #7 /(29783)

#9 ((randomized controlled trial or controlled clinical trial).pt. or randomized.ab. or placebo.ab. or clinical trials as topic.sh. or randomly.ab. or trial.ti.) not (animals not (humans and animals)).sh. /(1229328)

#10 #8 and #9 /(2181)

**3. Search strategy for Cochrane Central Register of Controlled Trials <**Issue 3 of 12, March 2019>

#1 MeSH descriptor [sepsis] explode all trees /(3970)

#2 MeSH descriptor [shock, septic] explode all trees /(763)

#3 steroid* in All Text /(25347)

#4 (sepsis in All Text or (septic in All Text and shock in All Text)) /(10284)

#5 #1 or #2 or #3 or #4 /(36517)

#6 MeSH descriptor [Adrenal Cortex Hormones] explode all trees /(13631)

#7 corticosteroid* in All Text /(16194)

#8 #6 or #7 /(26225)

#9 #5 and #8 in Trials /(5932)

**4. Search strategy for Latin American & Caribbean Health Sciences Literature,** http://www.bireme.br <March 22, 2019>

Search on: (sepsis or (septic shock)) and (glucocorticoid or corticosteroid or steroid) /(46)

**Supplement Method 2** Reason from exclusion of literature.

| **Author** | **Year** | **Reasons** |
| --- | --- | --- |
| Marik | 2019 | This is retrospective before-after study. |
| Kyriazopoulou | 2019 | This is a review. |
| Tilouche | 2019 | This is comparing between continuous and intermittent administration of hydrocortisone. |
| Hyvernat | 2018 | This is to study the difference in the benefits of different doses of hydrocortisone. |
| Kido | 2018 | This is a retrospective and observational study. |
| Marik | 2018 | This is a review. |
| Ngaosuwan | 2018 | This is to study the difference in the benefits of different doses of hydrocortisone. |
| Tongyoo | 2018 | This is a retrospective study from the previously reported randomized controlled trial.. |
| Antcliffe | 2018 | This is a retrospective study from the previously reported VANISH trial in 2014 and 2016. |
| Menon | 2017 | The participants were pediatric septic shock. |
| Ngaosuwan | 2017 | The intervention and control were 100 mg or 200 mg per day of hydrocortisone. |
| Billot | 2017 | This is a statistical analysis plan and did not meet the study design of this meta-analysis. |
| Gordon | 2016 | This was a factorial study exploring the early use of vasopressin versus noradrenalin and therefore the data are somewhat complex, and the population randomization was complex and likely to be mixed. |
| Maiden | 2016 | The intervention was Triiodothyronine. |
| Hyvernat | 2016 | The interventions were both low-dose hydrocortisone. |
| Annane | 2016 | This is a review. |
| Meduri | 2016 | This is a review. |
| O’Hearn | 2016 | This is a study protocol. |
| Chen | 2015 | The drugs of experimental and control group were same |
| Raghunathan | 2015 | The interventions were enteral fluid therapy. |
| Donnino | 2015 | The interventions were enteral ubiquinol. |
| Hung | 2015 | The participants were severely burned patients. |
| Póvoa | 2015 | The interventions were steroids, whether it's specifically cortisol or not is unclear. |
| Carmean | 2015 | This is a questionnaire survey. |
| Lipcsey | 2015 | This is a review. |
| Cho | 2015 | This is a review. |
| Venet | 2015 | This study mainly focused on severely burned patients. |
| Gordon | 2014 | The reason is that not all patients in the trial received hydrocortisone or placebo (50 of the 61 patients reported), and qualified data was not available. |
| Fink | 2014 | This is a review. |
| Marshall | 2014 | This is a review. |
| Sharma | 2014 | This is a study protocol. |
| Annane | 2013 | The intervention was drotrecogin alfa (activated). |
| Laviolle | 2013 | The participants were healthy volunteer. |
| Vincent | 2013 | This is a review. |
| Venkatesh | 2013 | This is a study protocol. |
| Hicks | 2012 | The subjects of this study were beagles. |
| Cherfan | 2011 | The intervention mainly focused on etomidate. |
| Huh | 2011 | The interventions of experimental and control group were same. |
| Deng | 2011 | The reported outcomes did not meet our outcomes. |
| Annane | 2010 | The intervention were intensive insulin infusion and conventional insulin therapy for patients treated with corticosteroids. |
| Salluh | 2010 | This was a review. |
| Valoor | 2009 | The participants were pediatric septic shock. |
| Cuthbertson | 2009 | The intervention mainly focused on etomidate. |
| Dellinger | 2009 | The intervention was phospholipid emulsion |
| Russell | 2009 | The intervention was vasopressin or norepinephrine. |
| Yeager | 2009 | The reported outcomes did not meet our outcomes. |
| Kaufman | 2008 | The effects of corticosteroids were assessed at baseline and after 24 h, and the reported outcomes mainly focused on neutrophil functions |
| Rinaldi | 2008 | The interventions were Vasopressin versus Norepinephrine. |
| Loisa | 2007 | The interventions were both low-dose hydrocortisone but with different adminstration of way. |
| Mikami | 2007 | The participants were diagnosed with community-acquired pneumonia without sepsis and septic shock |
| Cicarelli | 2006 | The participants were diagnosed with SIRS with or without sepsis, and the data of sepsis or septic shock was not available. |
| Levy | 2005 | The intervention was drotrecogin alfa. |
| Markovitz | 2005 | This was a retrospective cohort study. |
| Memis | 2004 | The intervention was lornoxicam. |
| Keh | 2003 | This study mainly focused on immunologic and hemodynamic results and did not provide data for outcomes in this meta-analysis. |
| Meduri | 1998 | The participants were diagnosed with ARDS and the data of sepsis or septic shock was not available. |
| Weiget | 1985 | The participants were mixed population and the data of sepsis or septic shock was not available. |
| Lucas | 1984 | This was a quasi-randomized controlled trial |
| Mckee | 1983 | The participants were mixed population and the data of sepsis or septic shock was not available. |
| Thompson | 1976 | The reported outcomes did not meet our outcomes. |
| Klastersky | 1971 | This was a quasi-randomized controlled trial |
| Wagner | 1955 | This was a quasi-randomized controlled trial |
| Hahn | 1951 | The reported outcomes did not meet our outcomes. |

**Supplementary Figure 1** Risk of bias assessment.


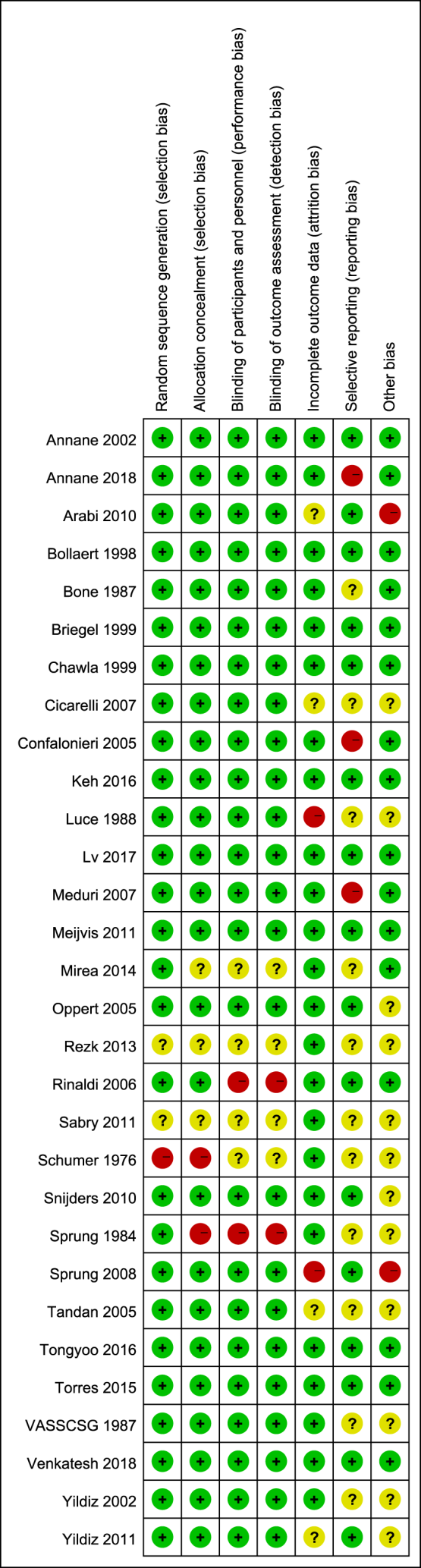


**Supplementary Figure 2** Meta-regression of the interaction of long course of low-dose corticosteroids in 28-day mortality and APACHE II score (A), mortality rate of baseline (B), the published year (C) and sample size (D).


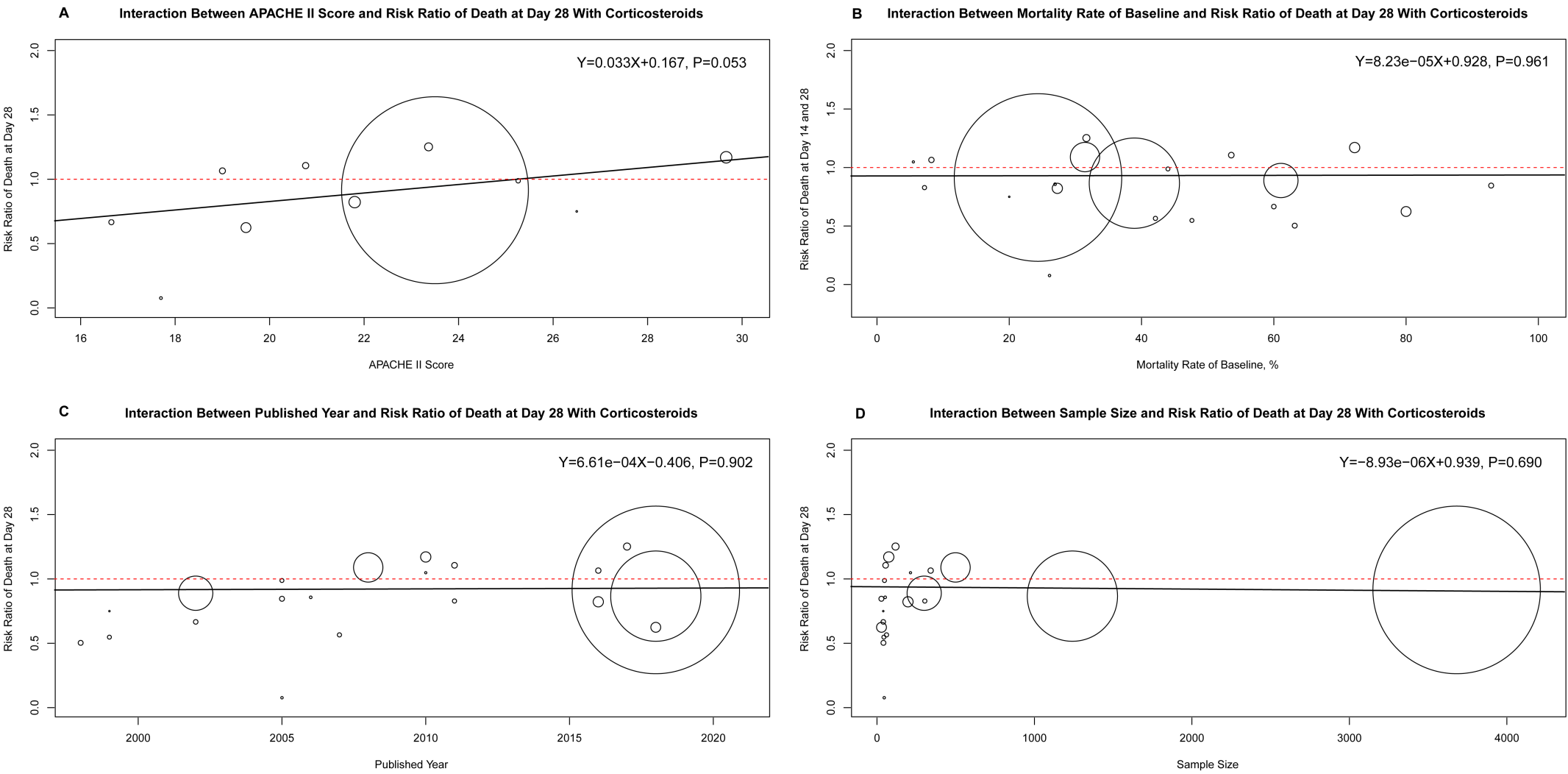


**Note:** Size of the data markers indicates weight of the study; APACHE, Acute Physiology and Chronic Health Evaluation.

**Supplementary Figure 3** Trial sequential analysis of long course of low-dose corticosteroids for 28-day mortality.


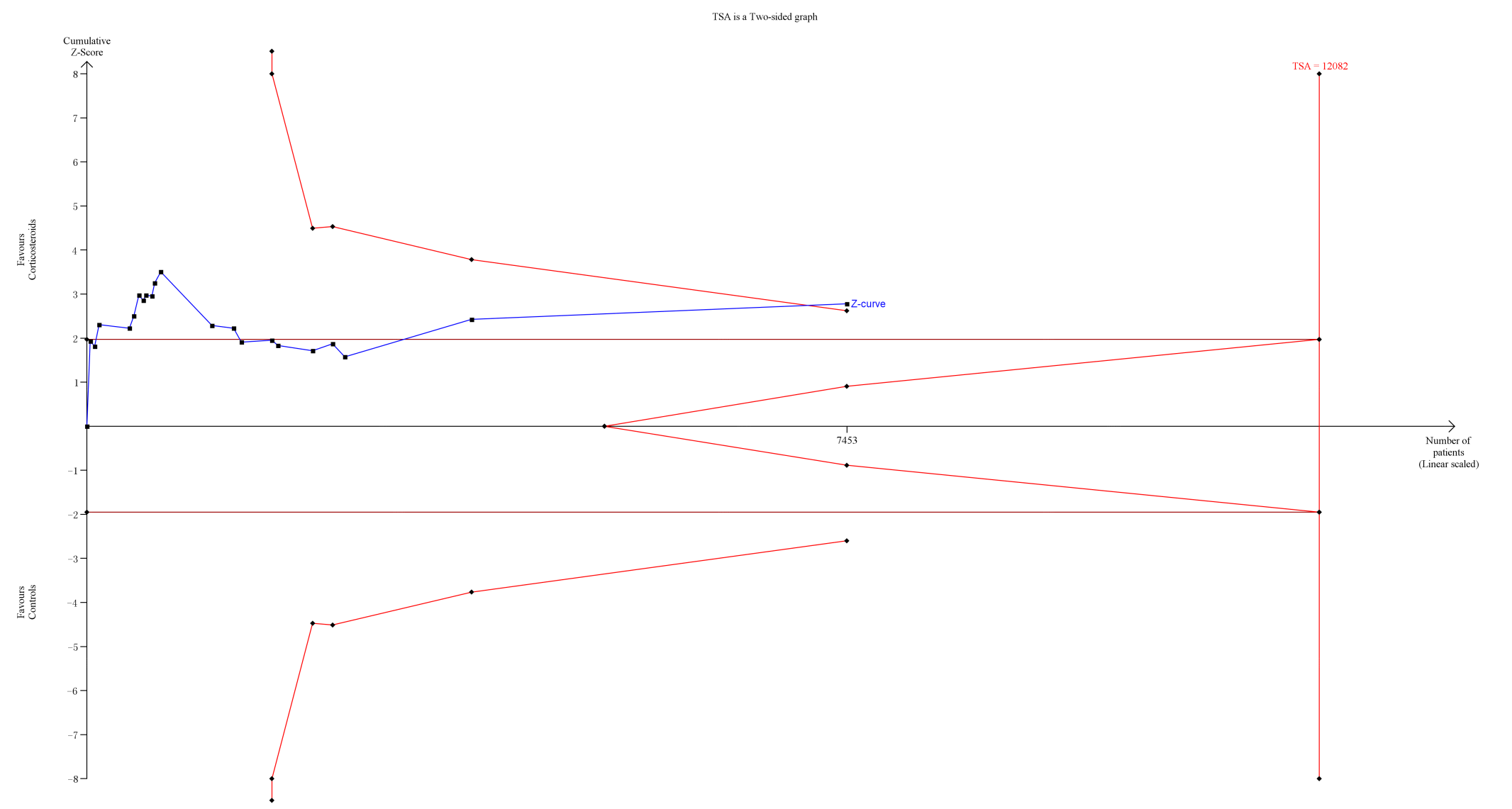


**Note:** A diversity-adjusted information size of 12,082 patients was calculated based on an anticipated relative risk reduction (RRR) of 10% (event proportion of 28.9% in the control arm, α=0.05 (two-sided), β=0.10 (Power 90%)). The blue cumulative Z-curve was constructed using a fixed-effects model and crossed the conventional boundaries and trial sequential monitoring boundaries when trials were included.

**Supplementary Figure 4** Trial sequential analysis of long course of low-dose corticosteroids for 28-day mortality as sensitive analysis.


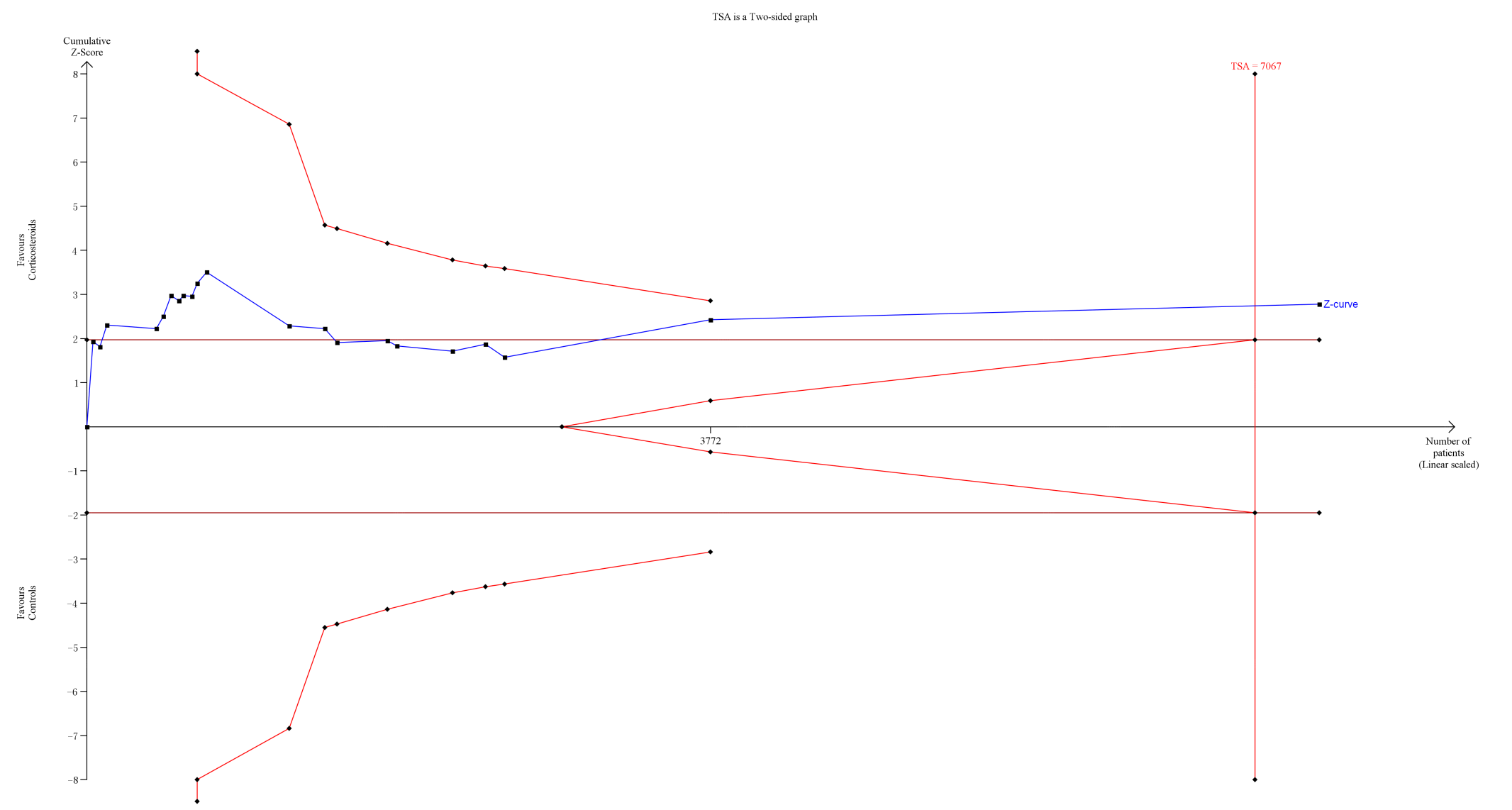


**Note:** A diversity-adjusted information size of 7,067 patients was calculated based on an anticipated relative risk reduction (RRR) of 13% (event proportion of 28.9% in the control arm, α=0.05 (two-sided), β=0.10 (Power 90%)). The blue cumulative Z-curve was constructed using a fixed-effects model and crossed the conventional boundaries and trial sequential monitoring boundaries when trials were included.

**Supplementary Figure 5** Trial sequential analysis of long course of low-dose corticosteroids for 28-day mortality as sensitive analysis.


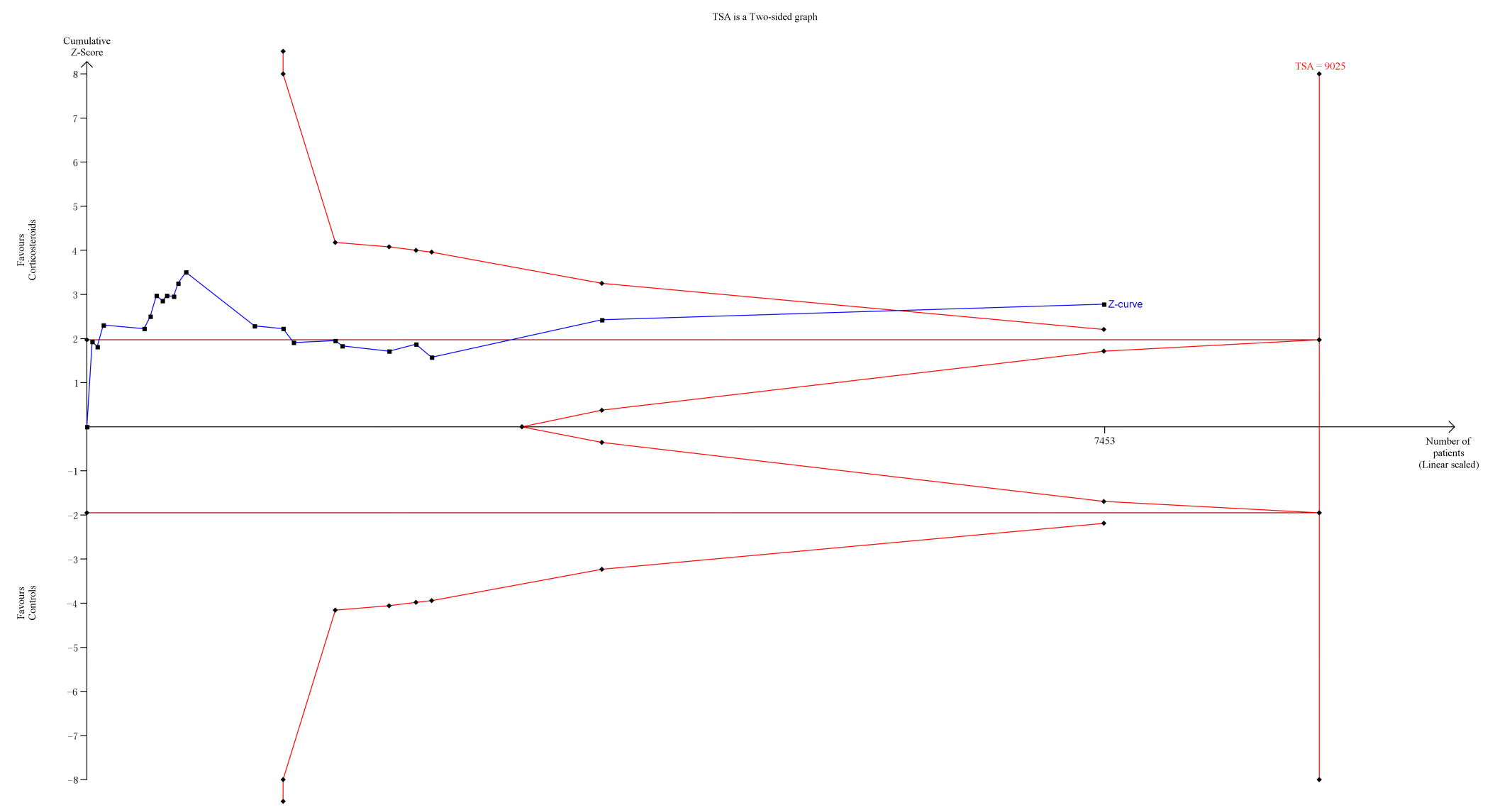


**Note:** A diversity-adjusted information size of 9,025 patients was calculated based on an anticipated relative risk reduction (RRR) of 10% (event proportion of 28.9% in the control arm, α=0.05 (two-sided), β=0.10 (Power 80%)). The blue cumulative Z-curve was constructed using a fixed-effects model and crossed the conventional boundaries but not trial sequential monitoring boundaries. However, the blue cumulative z-curve crossed the required information size (RIS) line when trials were included.

**Supplementary Figure 6** Trial sequential analysis of long course of low-dose corticosteroids for 90-day mortality.

**
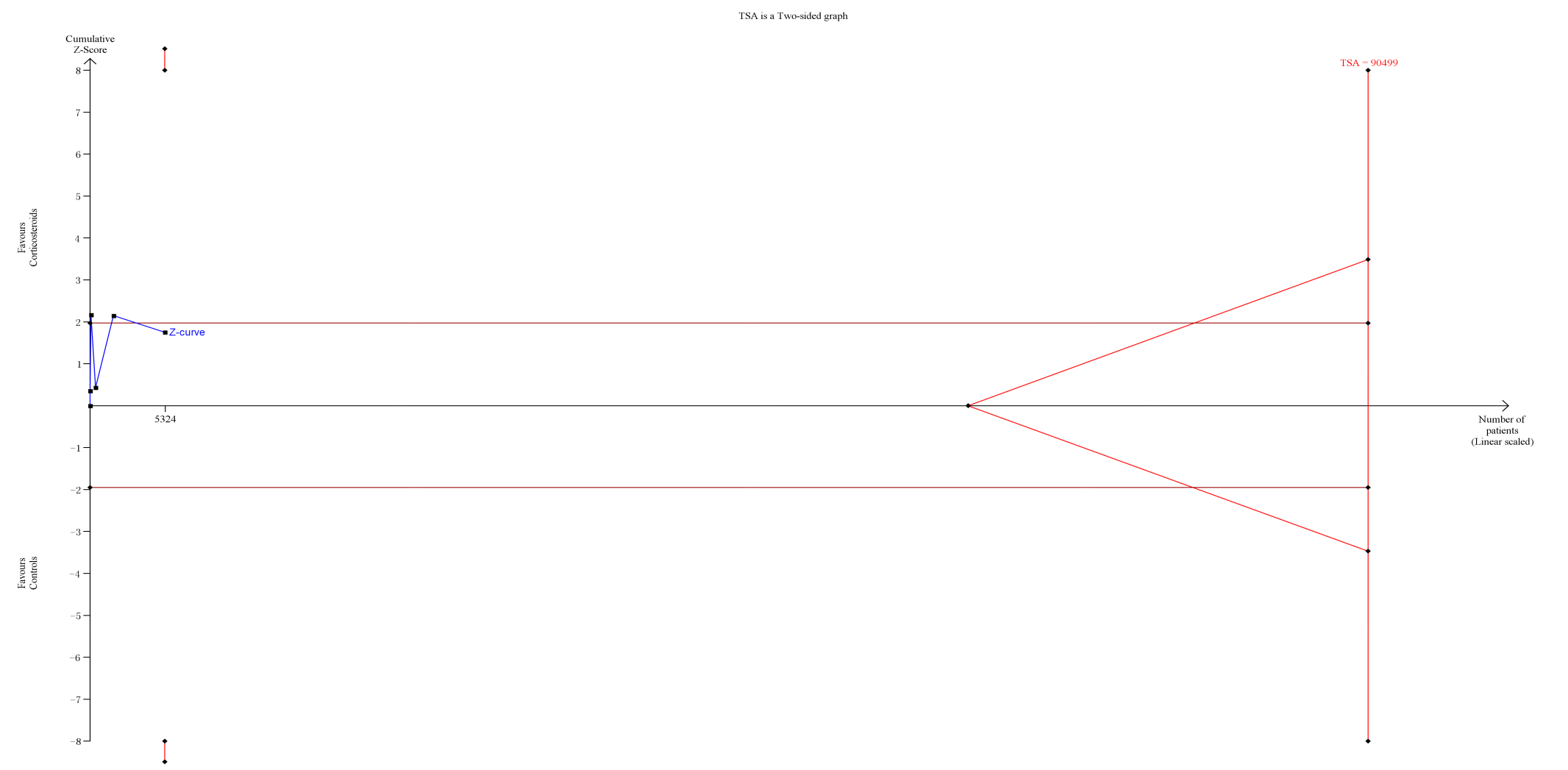
**

**Note:** A diversity-adjusted information size of 90,499 patients was calculated based on an anticipated relative risk reduction (RRR) of 10% (event proportion of 32.9% in the control arm, α=0.05 (two-sided), β=0.10 (Power 90%)). The blue cumulative Z-curve was constructed using a fixed-effects model and did not cross any boundaries when trials were included.

**Supplementary Figure 7** Trial sequential analysis of long course of low-dose corticosteroids for 90-day mortality as sensitive analysis.


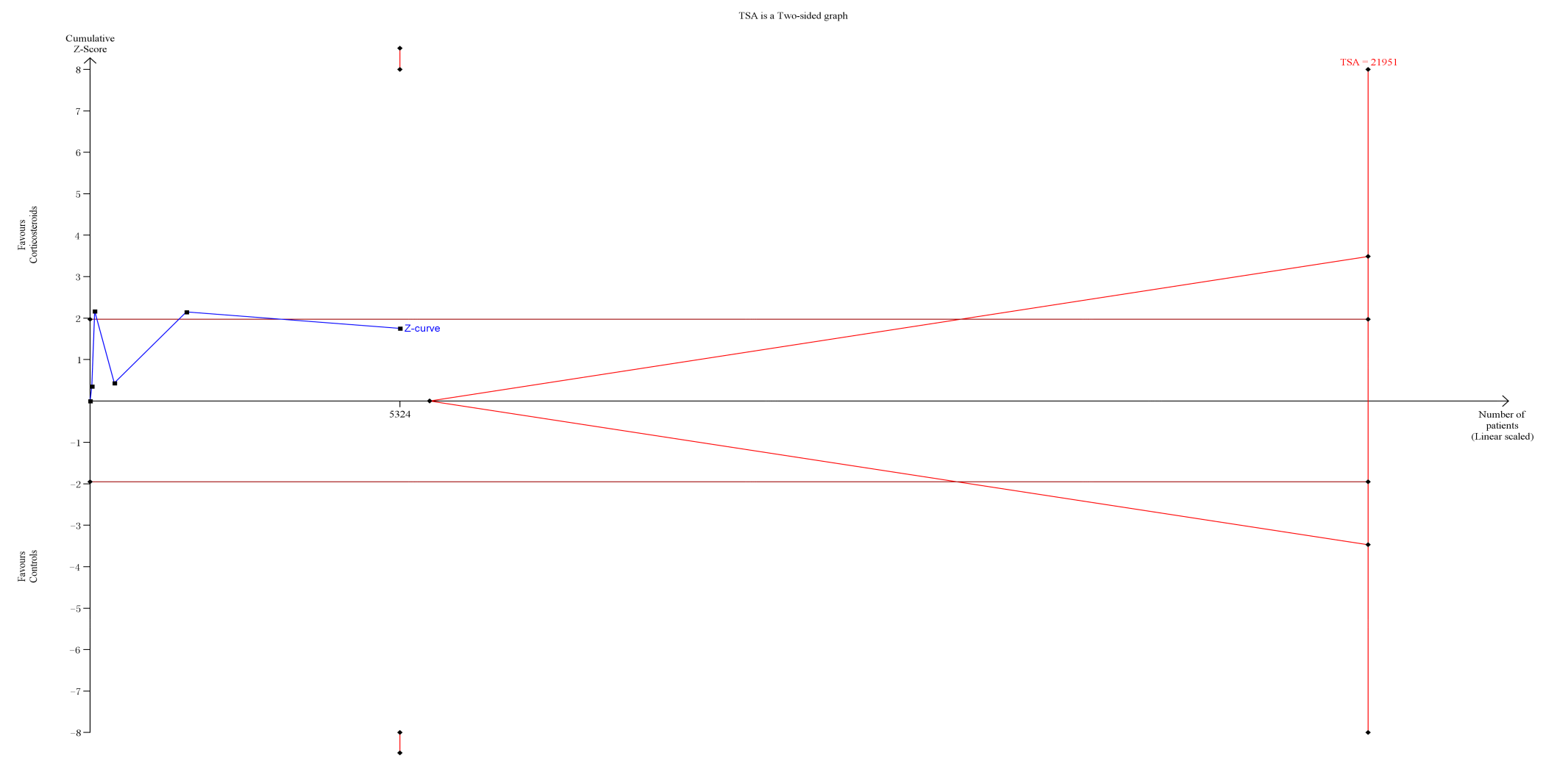


**Note:** A diversity-adjusted information size of 21,951 patients was calculated based on an anticipated relative risk reduction (RRR) of 20% (event proportion of 32.9% in the control arm, α=0.05 (two-sided), β=0.10 (Power 90%)). The blue cumulative Z-curve was constructed using a fixed-effects model and did not cross any boundaries when trials were included.

**Supplementary Figure 8** Trial sequential analysis of long course of low-dose corticosteroids for 90-day mortality as sensitive analysis.


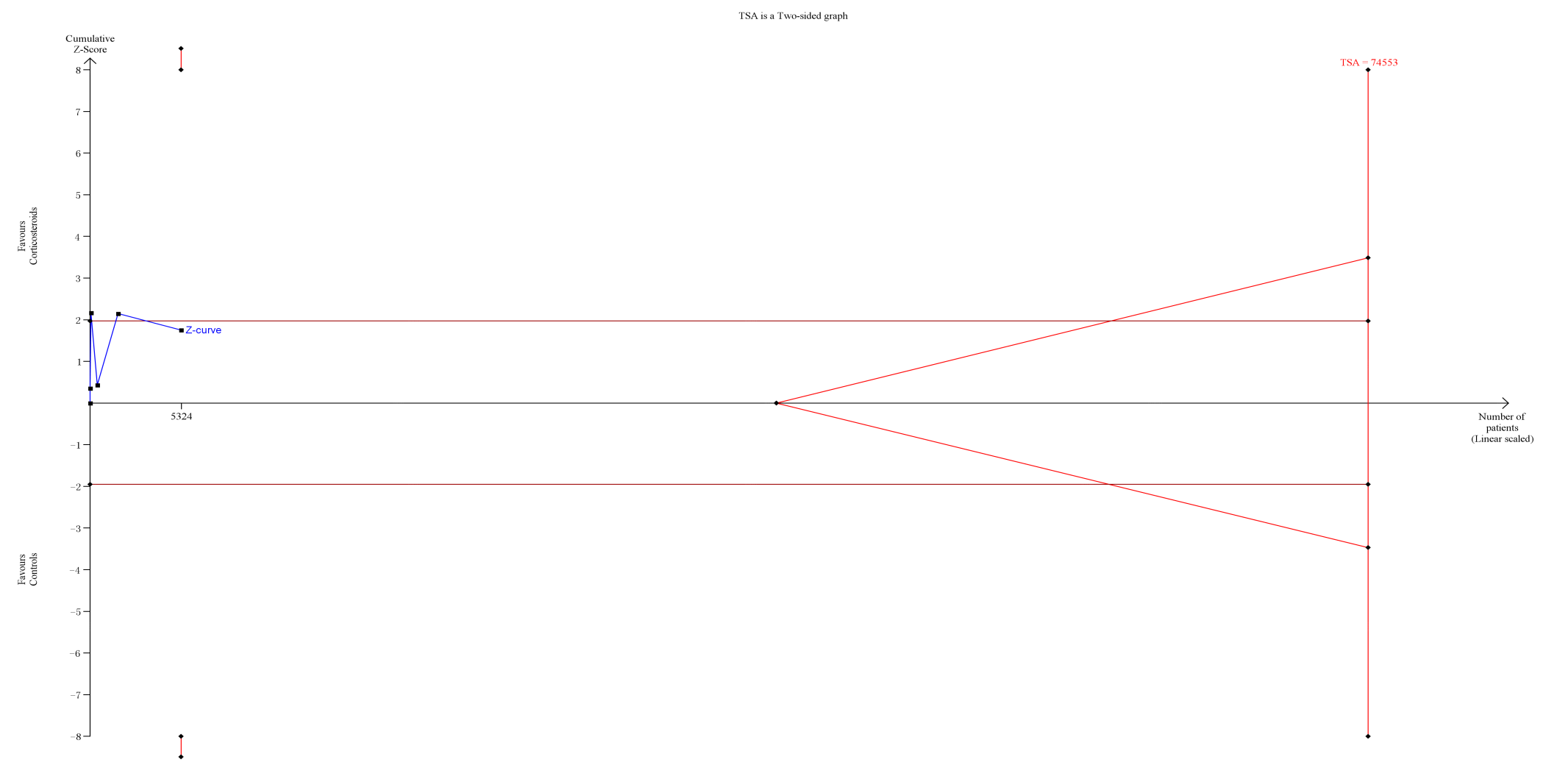


**Note:** A diversity-adjusted information size of 74,553 patients was calculated based on an anticipated relative risk reduction (RRR) of 10% (event proportion of 32.9% in the control arm, α=0.05 (two-sided), β=0.10 (Power 80%)). The blue cumulative Z-curve was constructed using a fixed-effects model and did not cross any boundaries when trials were included.

**Supplementary Figure 9** Forest plot of the benefits of long course of low-dose corticosteroids for ICU mortality.


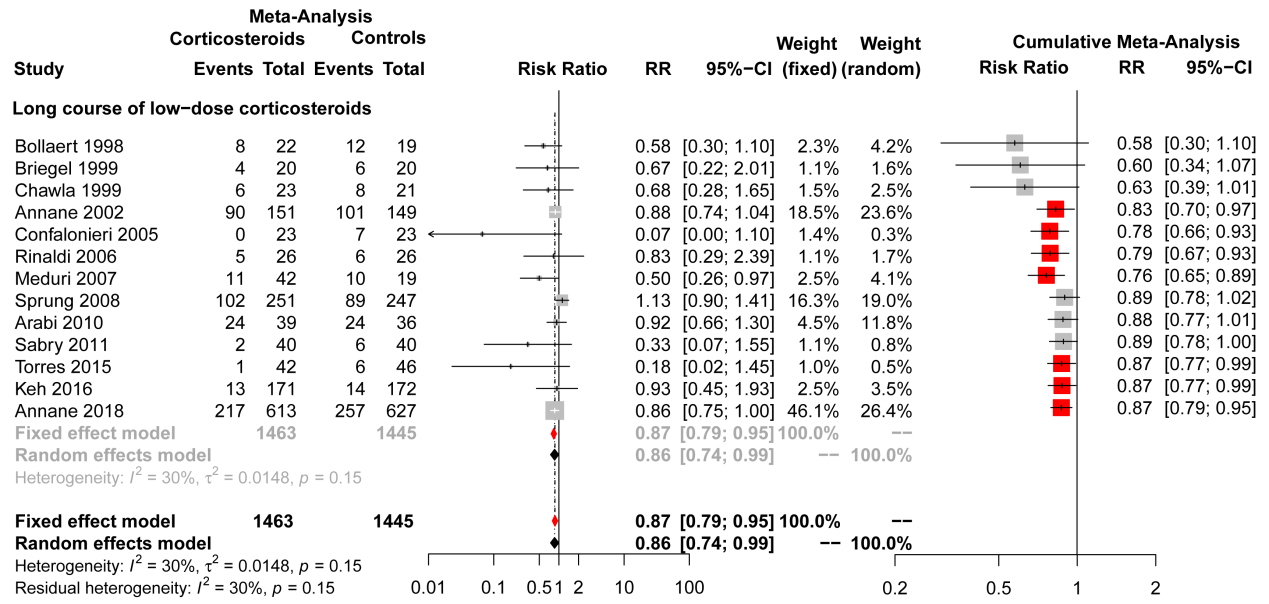


**Supplementary Figure 10** Trial sequential analysis of long course of low-dose corticosteroids for ICU mortality.


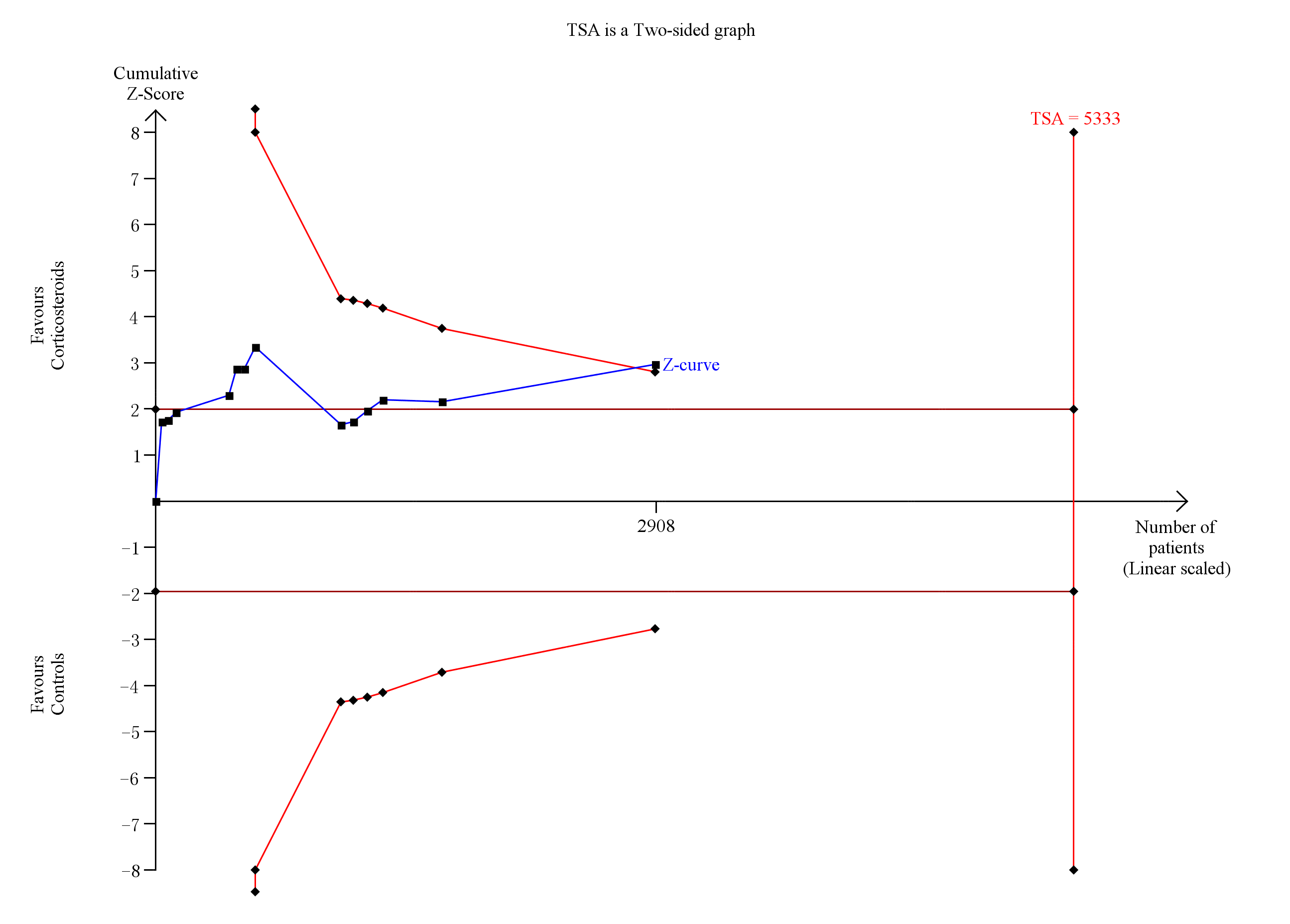


**Note:** A diversity-adjusted information size of 5,333 patients was calculated based on an anticipated relative risk reduction (RRR) of 18% (event proportion of 37.8% in the control arm, α=0.05 (two-sided), β=0.10 (Power 90%)). The blue cumulative Z-curve was constructed using a fixed-effects model and crossed the conventional boundaries and trial sequential monitoring boundaries when trials were included.

**Supplementary Figure 11** Trial sequential analysis of long course of low-dose corticosteroids for ICU mortality as sensitive analysis.


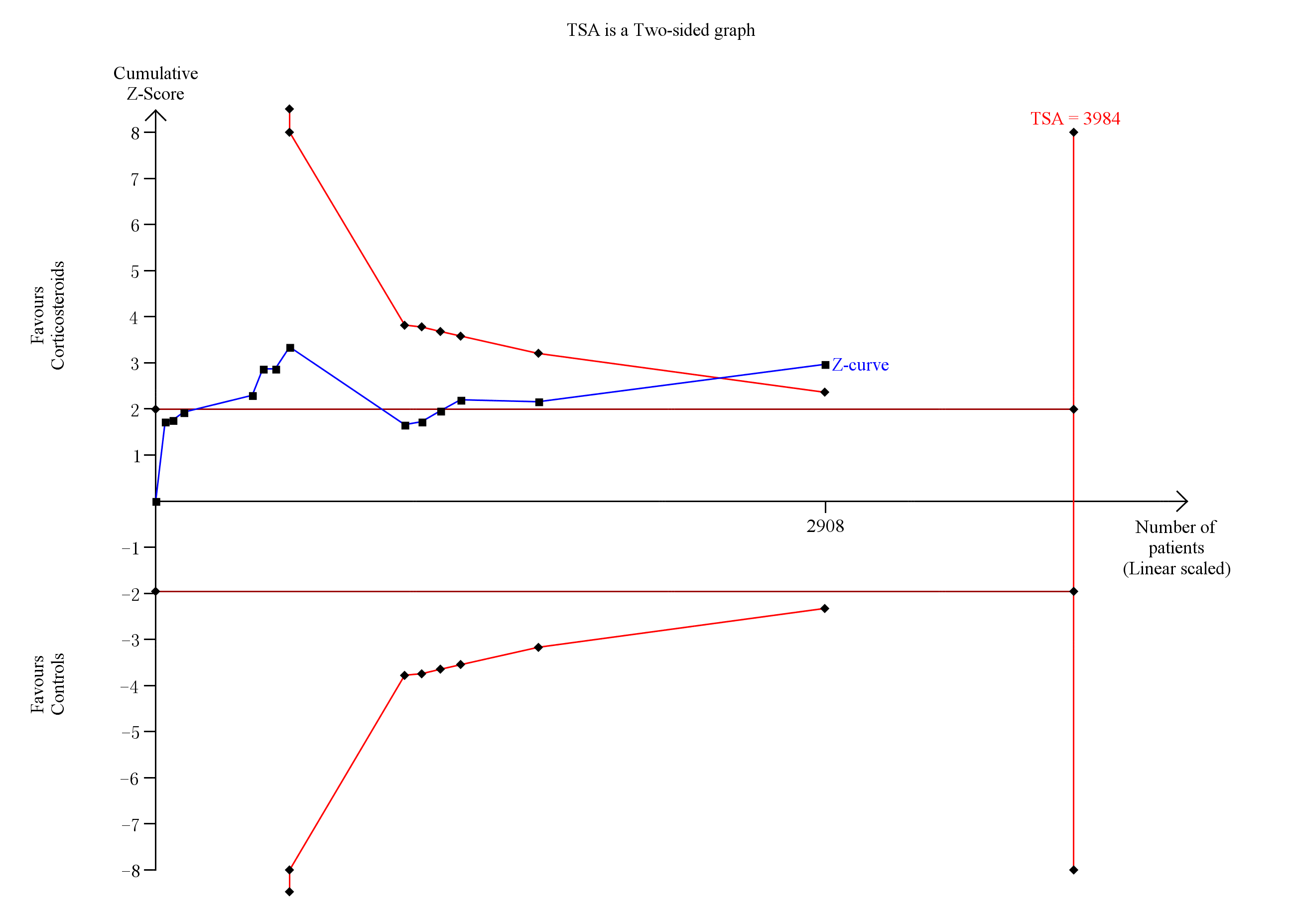


**Note:** A diversity-adjusted information size of 3,984 patients was calculated based on an anticipated relative risk reduction (RRR) of 18% (event proportion of 37.8% in the control arm, α=0.05 (two-sided), β=0.10 (Power 80%)). The blue cumulative Z-curve was constructed using a fixed-effects model and crossed the conventional boundaries and trial sequential monitoring boundaries when trials were included.

**Supplementary Figure 12** Forest plot of the benefits of short course of high-dose and long course of low-dose corticosteroids for in-hospital mortality.


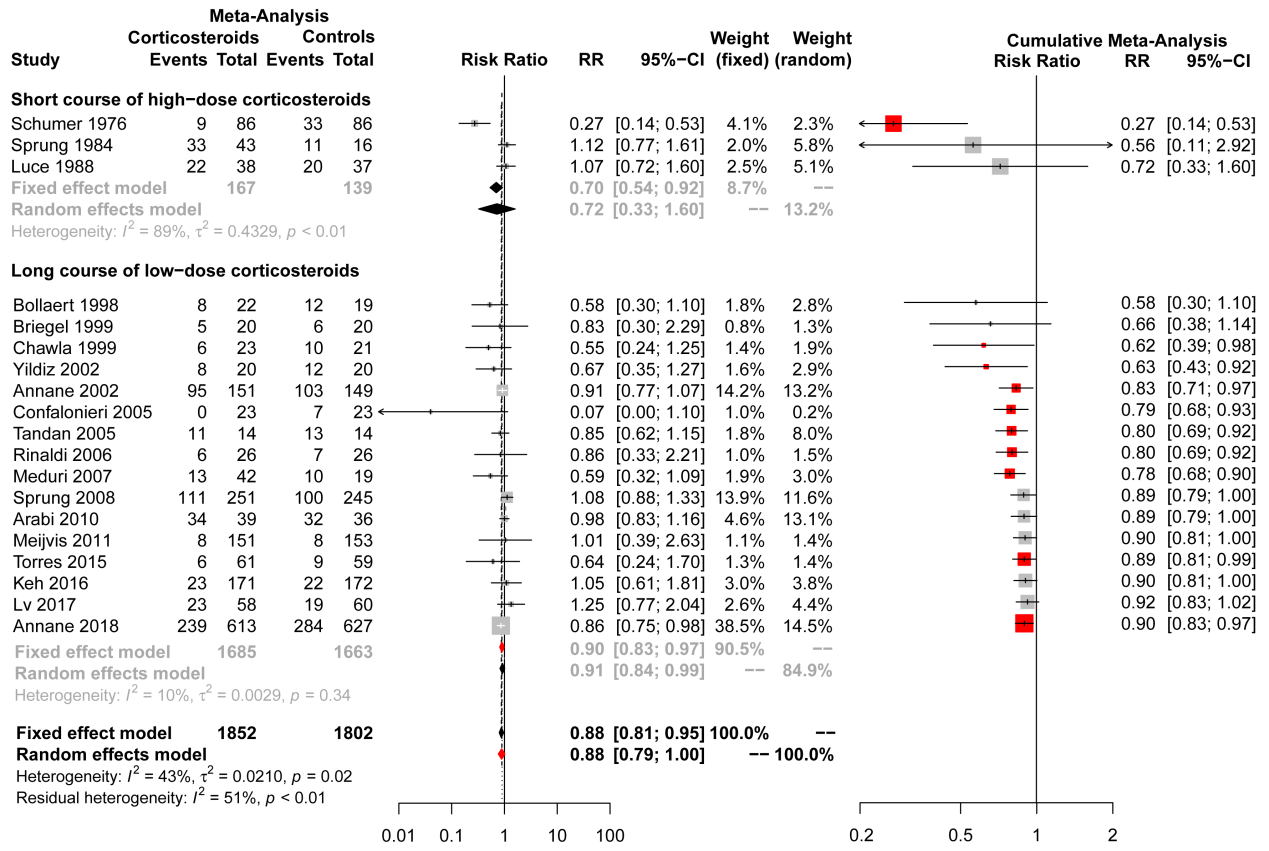


**Supplementary Figure 13** Trial sequential analysis of long course of low-dose corticosteroids for in-hospital mortality.


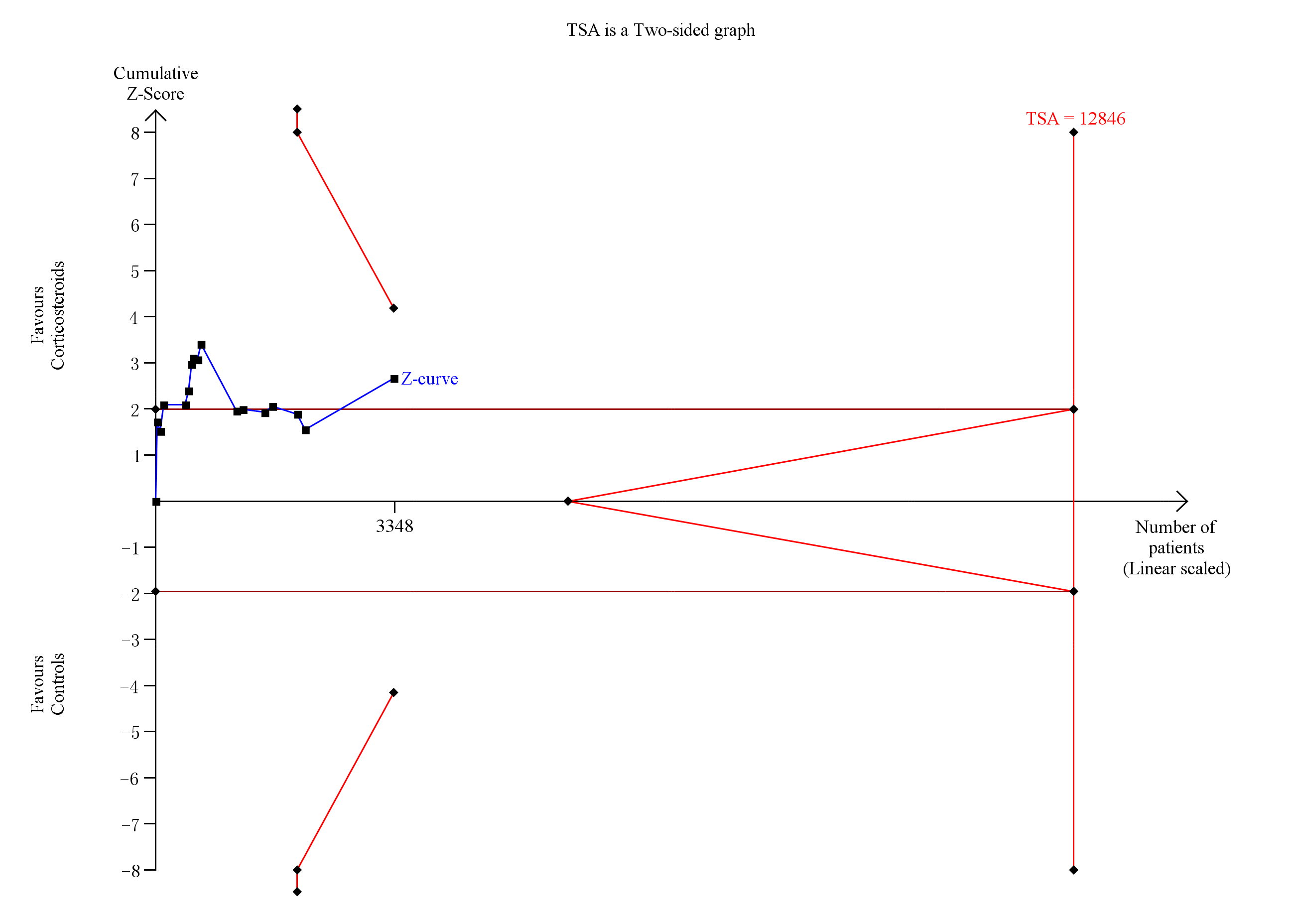


**Note:** A diversity-adjusted information size of 12,846 patients was calculated based on an anticipated relative risk reduction (RRR) of 9% (event proportion of 38.7% in the control arm, α=0.05 (two-sided), β=0.10 (Power 90%)). The blue cumulative Z-curve was constructed using a fixed-effects model and crossed the conventional boundaries and trial sequential monitoring boundaries when trials were included.

**Supplementary Figure 14** Trial sequential analysis of long course of low-dose corticosteroids for in-hospital mortality as sensitive analysis.


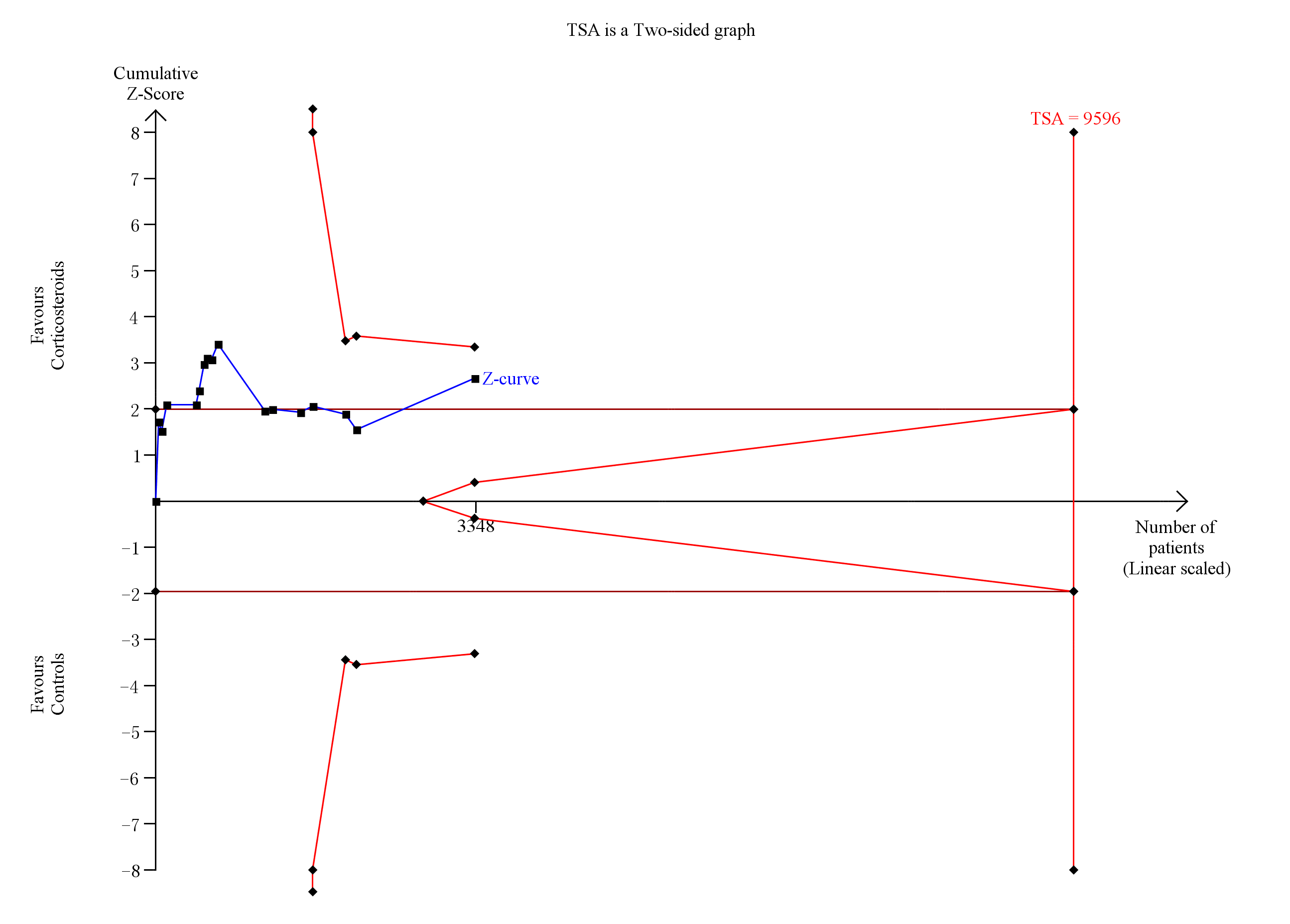


**Note:** A diversity-adjusted information size of 9,596 patients was calculated based on an anticipated relative risk reduction (RRR) of 9% (event proportion of 38.7% in the control arm, α=0.05 (two-sided), β=0.10 (Power 80%)). The blue cumulative Z-curve was constructed using a fixed-effects model and crossed the conventional boundaries and trial sequential monitoring boundaries when trials were included.

**Supplementary Figure 15** Forest plot of the benefits of short course of high-dose and long course of low-dose corticosteroids for shock reversal by day 7.


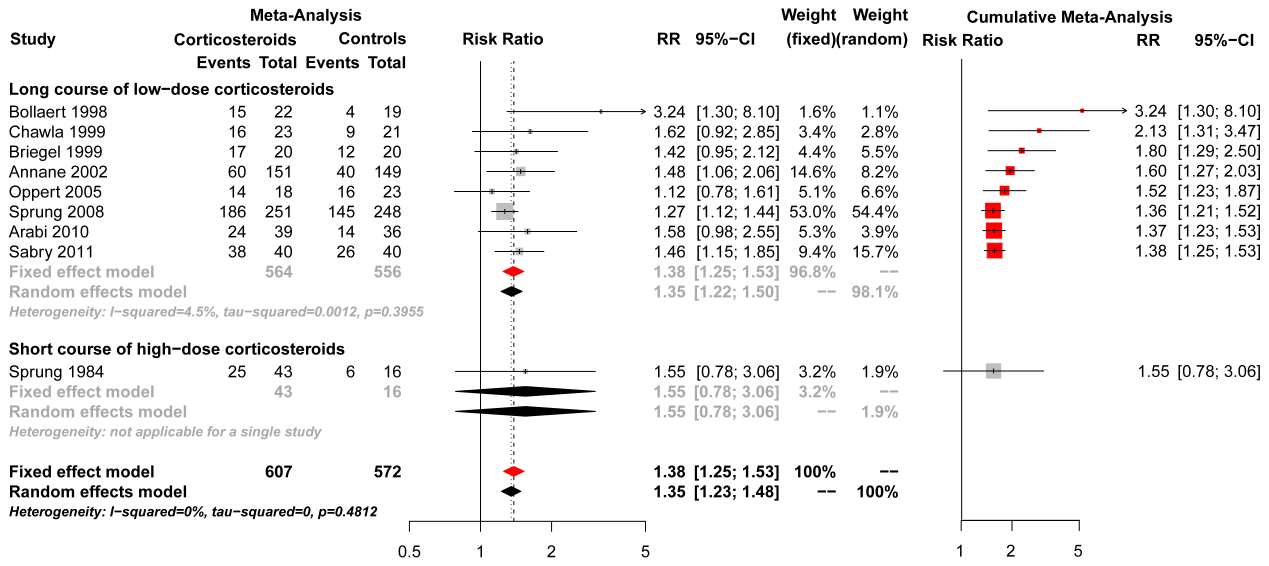


**Supplementary Figure 16** Forest plot of the benefits of long course of low-dose corticosteroids for shock reversal by day 28.

**
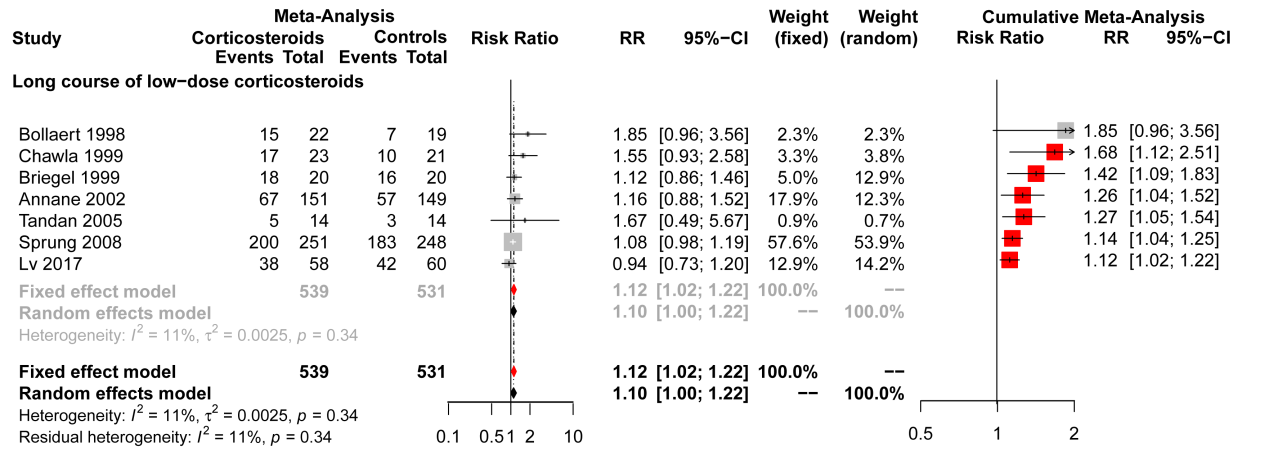
**

**Supplementary Figure 17** Forest plot of the benefits of long course of low-dose corticosteroids for length of ICU stay.

**
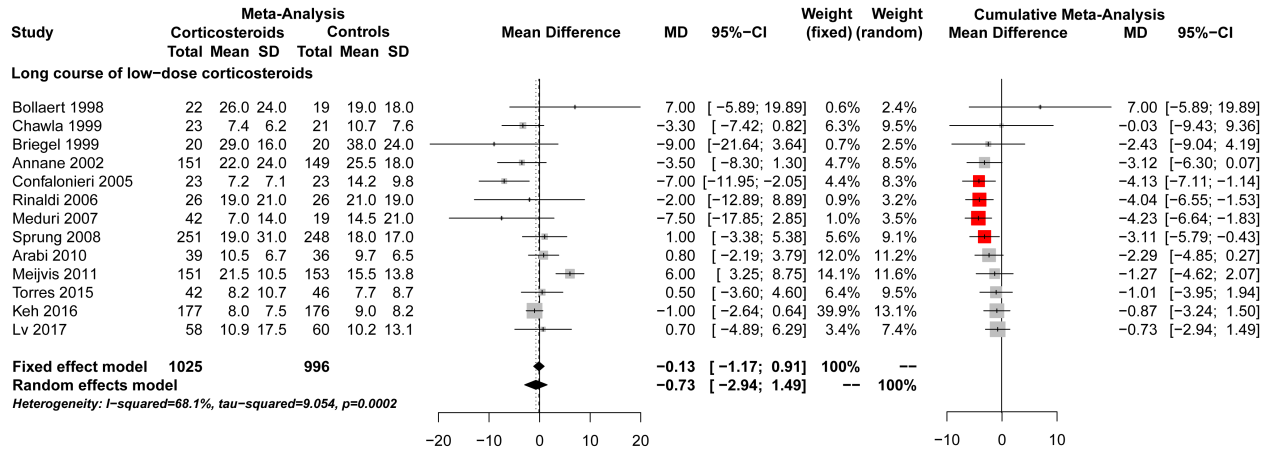
**

**Supplementary Figure 18** Forest plot of the benefits of long course of low-dose corticosteroids for length of hospital stay.

**
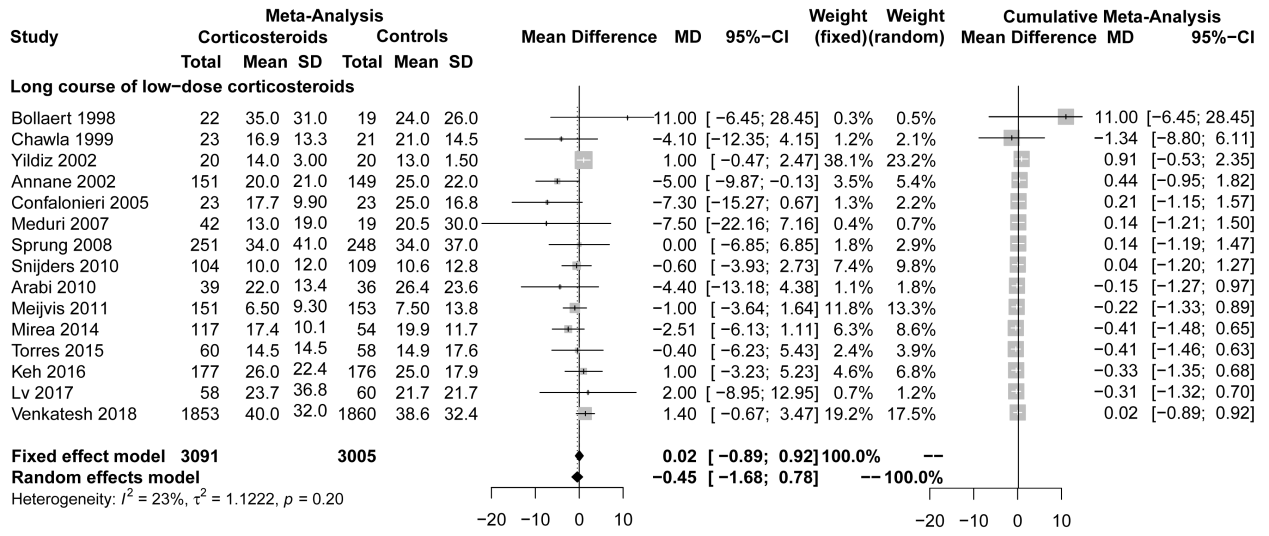
**

**Supplementary Figure 19** Forest plot of the benefits of long course of low-dose corticosteroids for SOFA score at day 7.

**
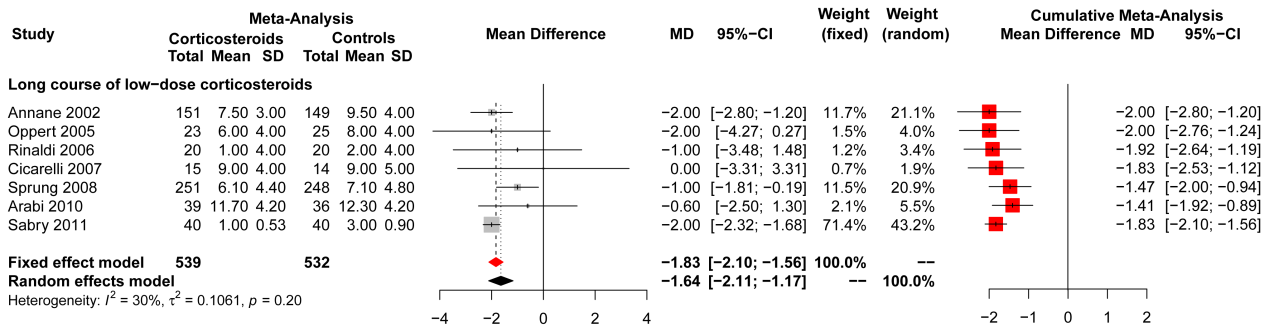
**

**Supplementary Figure 20** Forest plot of harms of short course of high-dose and long course of low-dose corticosteroids for gastroduodenal bleeding.

**
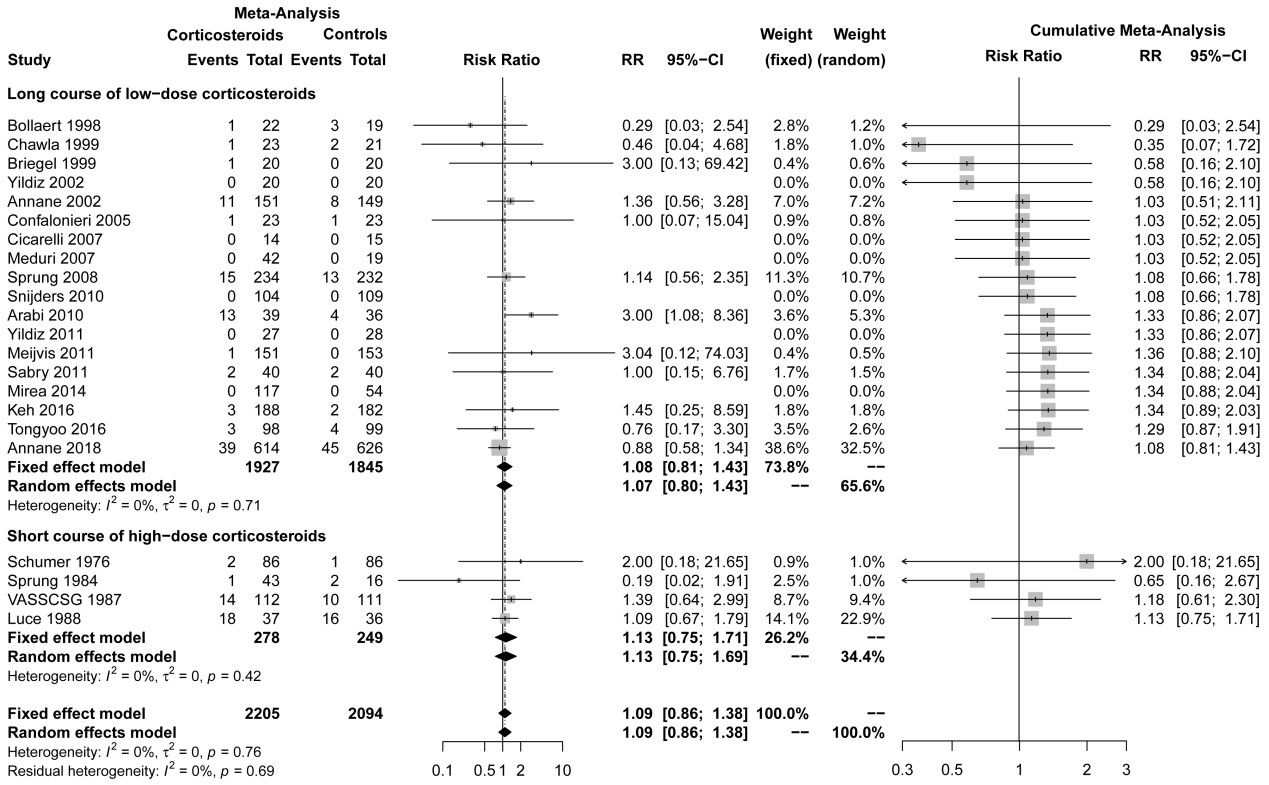
**

**Supplementary Figure 21** Forest plot of harms of short course of high-dose and long course of low-dose corticosteroids for superinfection.

**
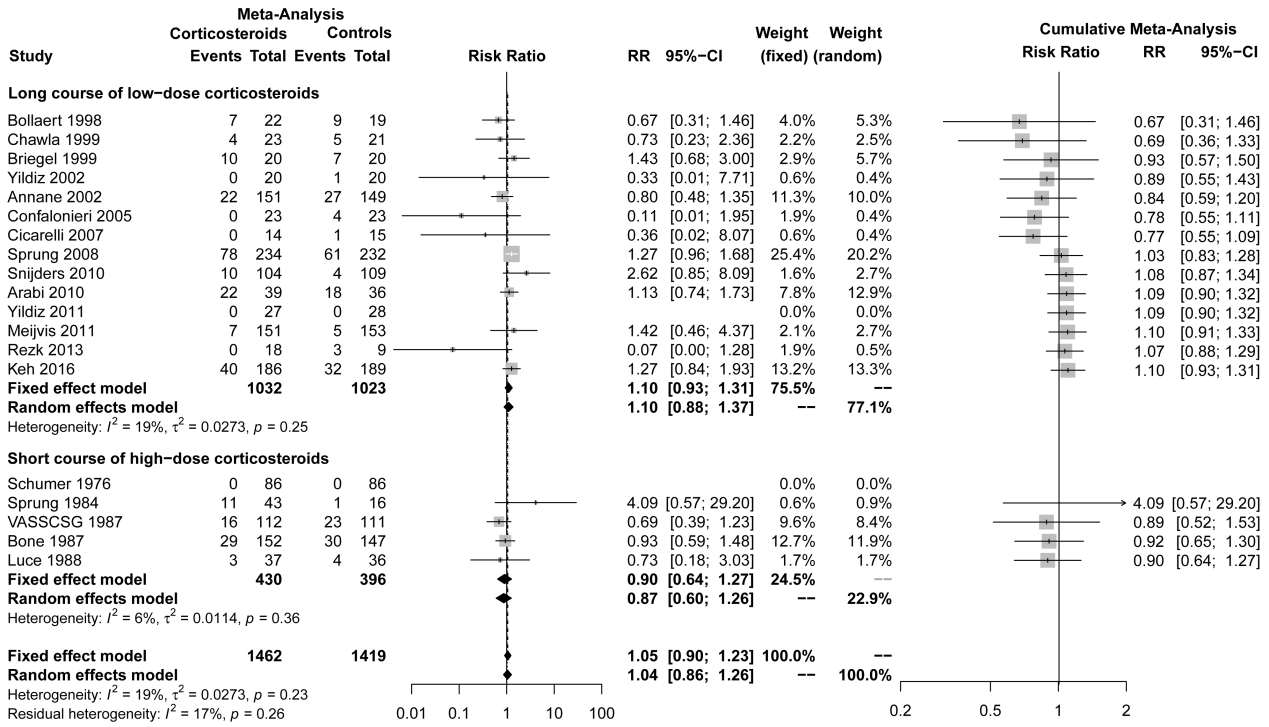
**

**Supplementary Figure 22** Forest plot of harms of long course of low-dose corticosteroids for neuromuscular weakness.

**
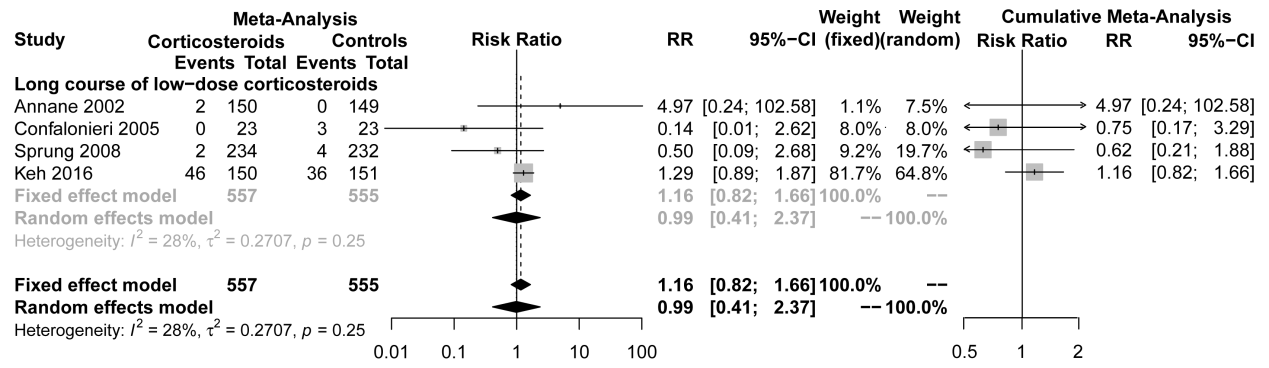
**

**Supplementary Figure 23** Forest plot of harms of short course of high-dose and long course of low-dose corticosteroids for hyperglycaemia.

**
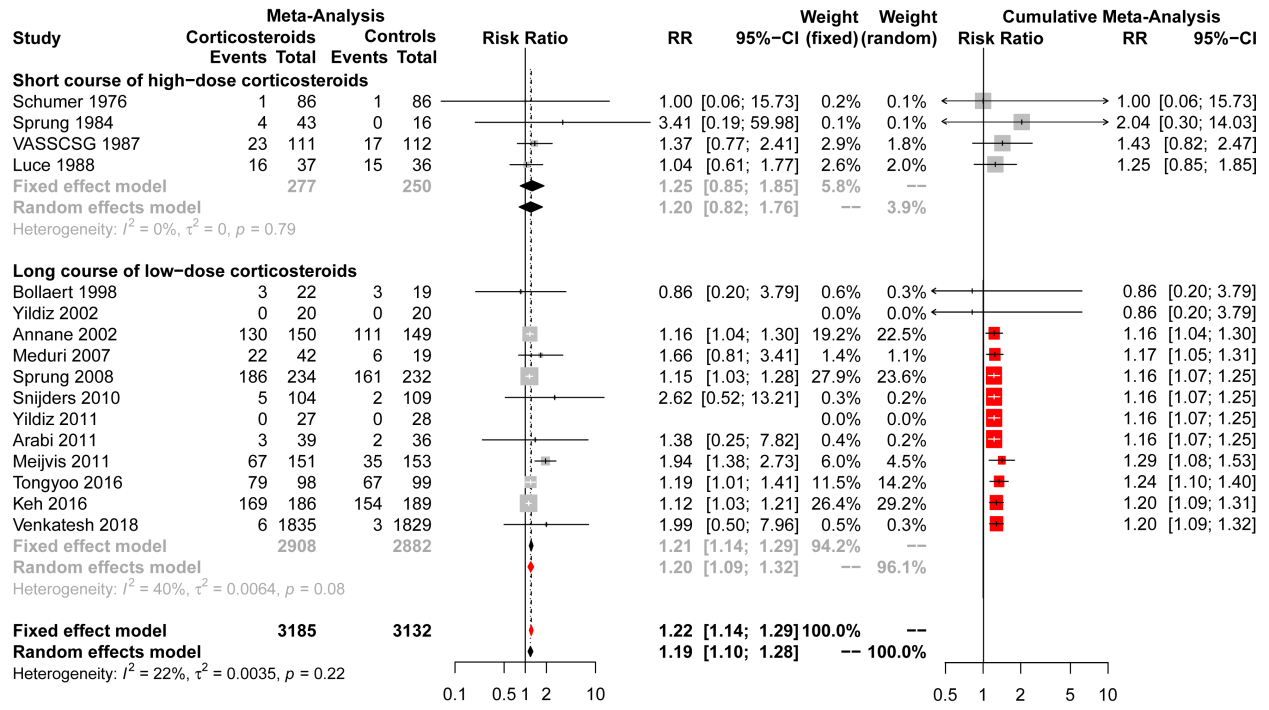
**

**Supplementary Figure 24** Forest plot of harms of long course of low-dose corticosteroids for hypernatraemia.

**
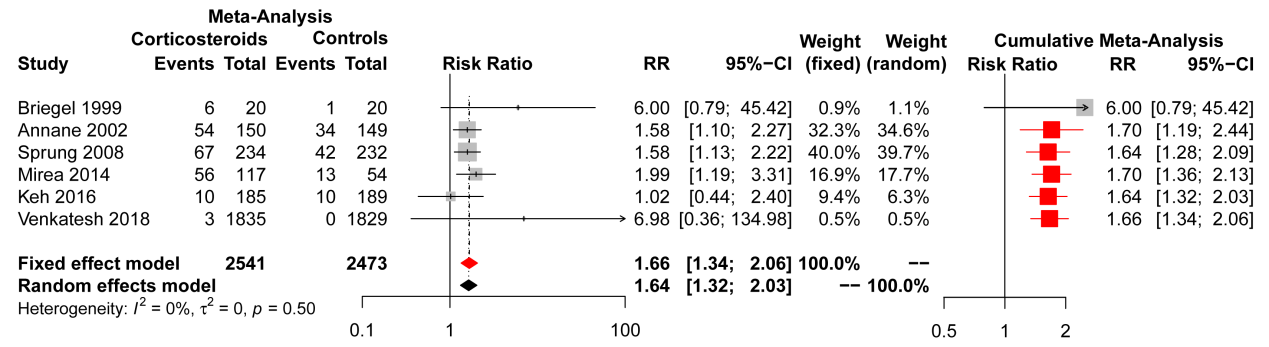
**

**Supplementary Figure 25** The L’Abbe plot and Egger’s regression of corticosteroids of long course of low-dose for 28-day mortality (A), 90-day mortality (B), ICU mortality (C) and hospital mortality (D).


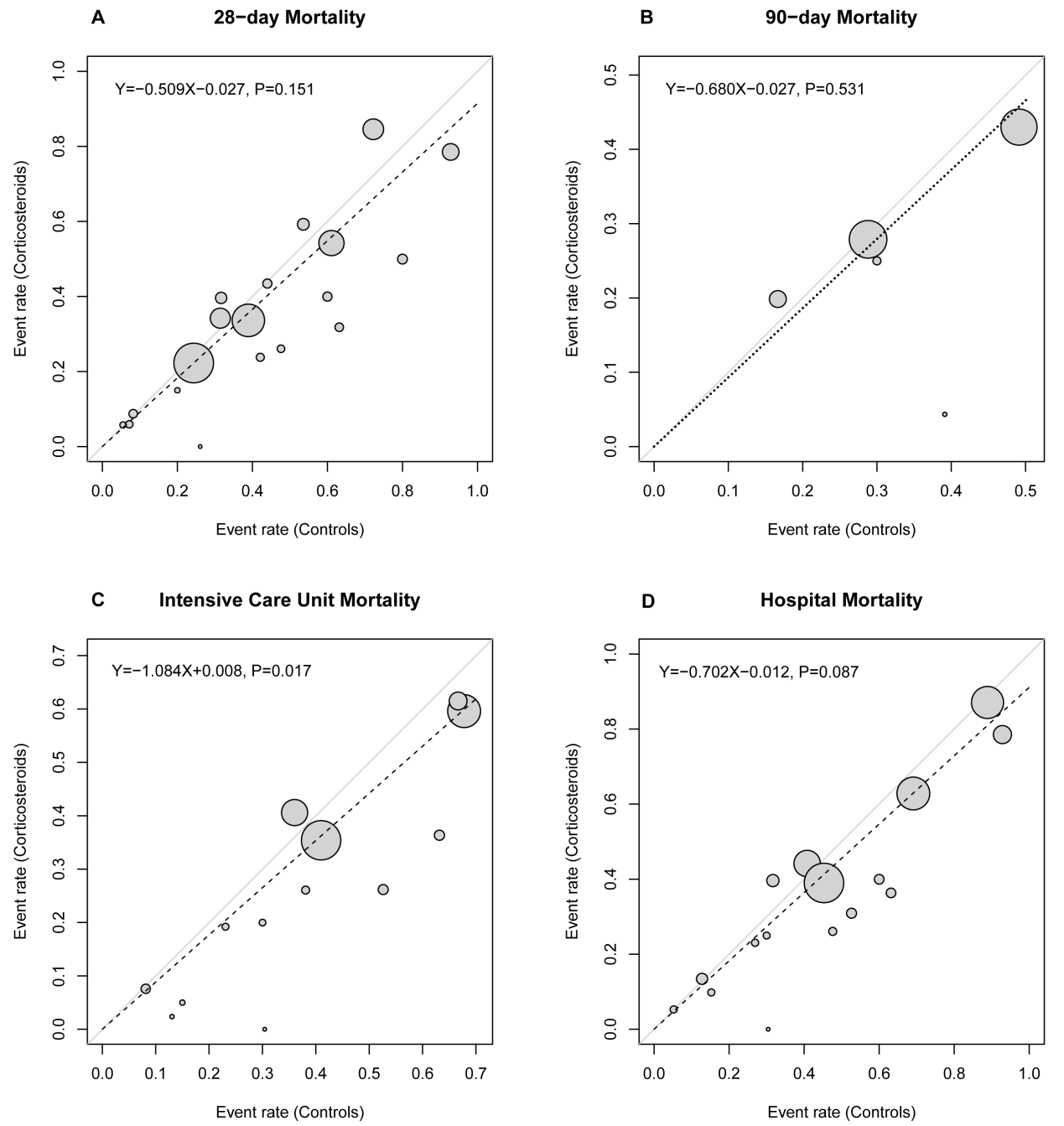

Supplement: Supplementary file 1 [file DataSheet_1.docx]
